# Microvascular dysfunction across organs in heart failure with preserved ejection fraction: the PROSE-HFpEF case-control study

**DOI:** 10.1186/s12933-025-02850-1

**Published:** 2025-07-30

**Authors:** Jerremy Weerts, Blanche L. M. Schroen, Arantxa Barandiarán Aizpurua, Tos T. J. M. Berendschot, Lloyd Brandts, Carroll A. B. Webers, Sami O. Simons, Steven J. R. Meex, Ronald Henry, Carla J. H. van der Kallen, Hans-Peter Brunner-La Rocca, Christian Knackstedt, Stephane R. B. Heymans, Rudolf A. de Boer, Vanessa P. M. van Empel, Alfons J. H. M. Houben

**Affiliations:** 1 Department of Cardiology, CARIM Cardiovascular Research Institute Maastricht, Maastricht University Medical Centre (MUMC+), PO Box 616, 6200 MD Maastricht, The Netherlands; 2 Department of Internal Medicine, CARIM Cardiovascular Research Institute Maastricht, Maastricht University Medical Centre (MUMC+), Maastricht, The Netherlands; 3 University Eye Clinic Maastricht, Maastricht University Medical Centre (MUMC+), Maastricht, The Netherlands; 4 Department of Respiratory Medicine, NUTRIM Research Institute of Nutrition and Translational Research in Metabolism, Maastricht University Medical Centre (MUMC+), Maastricht, The Netherlands; 5 Department of Clinical Chemistry, CARIM School for Cardiovascular Diseases, Maastricht University Medical Centre (MUMC+), Maastricht, The Netherlands; 6 Department of Cardiology, Thorax Center, Cardiovascular Institute, Erasmus MC, Rotterdam, the Netherlands; 7 Department of Clinical Epidemiology and Medical Technology Assessment, Maastricht University Medical Centre (MUMC+), Maastricht, The Netherlands

**Keywords:** Heart failure with preserved ejection fraction, Diastolic heart failure, Microcirculation, Pathophysiology, Sex differences, HFpEF, Microvascular dysfunction

## Abstract

**Background:**

Systemic microvascular dysfunction is proposed as a key pathophysiological process in heart failure with preserved ejection fraction (HFpEF). This study compared microvasculature across vascular beds in HFpEF patients and controls.

**Methods:**

This prospective, case-control study included subjects ≥ 60years. HFpEF patients were diagnosed in an expert centre. Controls without HF were selected from the Maastricht Study, a population cohort enriched with diabetes mellitus. Microvascular assessments included central retinal venular/arteriolar calibres (CRVE/CRAE), flicker-light-induced retinal dilation, skin microvascular flowmotion and heat-induced hyperemia, and urinary albumin-to-creatinine ratio (UACR). Group differences were evaluated with confounder-adjustments (age, sex, blood pressure, body mass index, diabetes, haemoglobin, smoking). Interactions with sex and diabetes mellitus were tested, and stratified analyses were performed when significant interactions were present.

**Results:**

Microvascular assessments were performed in 138 HFpEF patients and 2140 controls. Microvascular differences were present between groups in all vascular beds. However, confounder-adjusted analyses attenuated differences. Confounder-adjusted analyses indicated that HFpEF patients versus controls still had retinal differences: narrower CRVE (− 8.1 μm, *p* = 0.008) and narrower CRAE trend (− 3.5 μm, *p* = 0.073), but similar flicker-light-induced retinal venular/arteriolar dilation (− 0.23%, *p* = 0.392; − 0.18%, *p* = 0.593, respectively). Confounder-adjusted analyses showed similar skin flowmotion measures (i.e. endothelial power − 0.09log(PU^2^), *p* = 0.181), and heat-induced hyperemia (0.02log(%), *p* = 0.605) between groups. UACR remained higher in HFpEF after confounder adjustments (0.56log(g/mol), *p* = < 0.001). Interaction analyses revealed that female patients had narrower CRVE versus controls (p_int_=0.023; females − 13.8 μm, *p* < 0.001; males 1.2 μm, *p* = 0.812). Patients had lower skin endothelial flowmotion power only when diabetes was co-occurring (p_int_=0.048; − 0.36 log(PU^2^ ), *p* = 0.014). UACR was higher in male and female patients versus controls, but was more pronounced in males (p_int_=0.002).

**Conclusions:**

HFpEF patients showed microvascular differences versus controls across all vascular beds studied. However, confounder-adjusted differences remained significant in eyes and kidneys. The findings across multiple organs support that MVD is likely a more systemic process than only local MVD in HFpEF, and possible sex-specific underlying pathophysiology.

**Registration:**

URL: https://onderzoekmetmensen.nl; Unique identifier: NL7655.

**Graphical abstract:**

**Figure:**
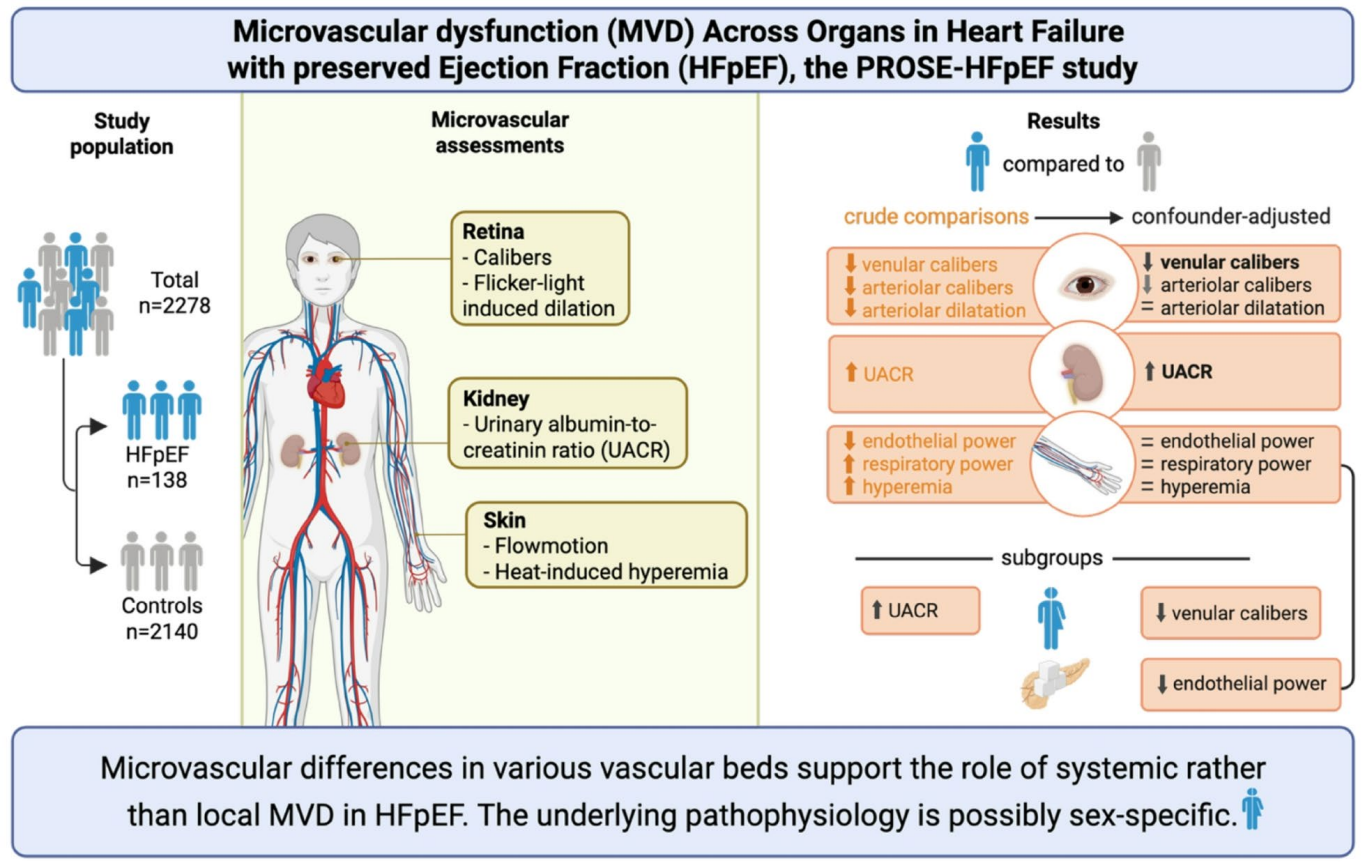
Figure created in BioRender (i88u523).

**Supplementary Information:**

The online version contains supplementary material available at 10.1186/s12933-025-02850-1.

## Research insights

**What is currently known about this topic?**

- Systemic microvascular dysfunction (MVD) is proposed a key pathophysiological process in HFpEF, but human data are limited.

**What is the key research question?**

- Is MVD systemically present in patients with HFpEF?

**What is new?**

1. HFpEF patients compared to controls showed systemic microvascular alterations, i.e. across all vascular beds studied.
2. After confounder-adjustments, only microvascular beds of the eyes and kidneys remained significantly different.
3. The findings appeared different between females and males and hint at other pathomechanisms than only NO-dependent MVD.

**How might this study influence clinical practice?**

- Markers of MVD may facilitate HFpEF screening, risk-stratification, and targeted therapies, warranting further longitudinal studies.

## Background

Heart failure with preserved ejection fraction (HFpEF) is a prevalent syndrome with a high symptom burden and mortality rate, yet its underlying mechanisms remain poorly understood. Emerging evidence identifies microvascular dysfunction (MVD) as a potential key driver for the multi-organ HFpEF syndrome [1]. Several mechanisms can induce MVD in HFpEF, of which chronic systemic inflammation due to comorbidities (e.g. hypertension, obesity, diabetes mellitus (DM), and anaemia) has been reported most [2, 3]. Consequently, MVD can lead to systemic end-organ damage in cardiac and extra-cardiac tissues [1]. While preclinical studies support this mechanistic hypothesis, human data remain limited [4]. Rather, most studies focused on endothelial function in larger vessels than actual microcirculation [1]. Also, cardiovascular risk factors can drive MVD independently of HFpEF development, and therefore, adequate correction for these factors is crucial to dissect the independent contribution of MVD to HFpEF, which was lacking in previous studies [1]. 

Understanding sex-specific differences in HFpEF pathophysiology, including the role of MVD, is important given the disproportionate female predisposition for transitioning from preclinical diastolic dysfunction to HFpEF [5]. Understanding the underlying disease mechanisms may be essential to develop new diagnostic tools or effective interventions that could be sex-specific.

The Prospective Case-Control Study to Evaluate Systemic Microvascular Dysfunction in Heart Failure with Preserved Ejection Fraction in Males and Females (PROSE-HFpEF) was carried out to assess the role of MVD in HFpEF. This study evaluated multiple aspects of MVD across vascular beds in patients diagnosed with HFpEF and population-based control individuals, carefully adjusted for confounders and exploring sex-specific differences.

## Methods

### Study design and population

This cross-sectional, case-control study included participants aged ≥ 60 years who underwent standardised microvascular assessments and preparations per Standard Operating Procedures, as detailed previously [6] (Supplemental Methods), to identify systemic microvascular differences between patients with HFpEF and control individuals after correction for confounders. The study reports according to the STROBE recommendations.

Patients with HFpEF were recruited between April 2019 and June 2021 from a dedicated clinic that systematically evaluated HFpEF diagnosis using echocardiography, natriuretic peptides, and on indication right heart catheterisation (detailed previously [7, 8]). HFpEF diagnosis was according to the European Society of Cardiology (ESC) HF 2016 guidelines (Supplemental Methods). Patients with contraindications for ocular drip preparation needed for retinal assessments were excluded.

Control individuals were recruited from The Maastricht Study, a population-based cohort enriched with individuals with type 2 DM [6, 9]. They were selected based on the availability of flicker-light-induced retinal arteriolar dilation results. Control individuals were excluded if they had HF at baseline or within one-year follow-up, or suspected severe cardiac valve disease or reduced left ventricular (LV) ejection fraction on baseline echocardiography. All inclusion and exclusion criteria are detailed in Supplemental Methods.

All subjects provided written informed consent. The study was conducted following the Declaration of Helsinki and approved by an independent ethics committee (METC19-005). It was also pre-registered at the Netherlands Trial Register (NTR NL7655). The Supplemental Methods provide the sample size calculations and protocol deviations due to the Coronavirus Disease-19 (COVID-19) pandemic (mainly premature inclusion closure).

### Microvascular assessments

We evaluated differences in microvascular functions between HFpEF patients and controls in retinal, skin, and renal vascular beds, adjusting for key confounders of microvascular function and HFpEF in an identical setting [1]. The microvascular assessments have been selected because they have previously been associated with incident HF or were reported different in HFpEF versus controls (in smaller studies), and can all be altered dynamically (i.e. by factors such as hyperglycaemia, NO-bioavailability, and inflammation) [1, 10]. All microvascular assessments have been detailed elsewhere before [6]are provided in Supplemental Methods, and are briefly summarised below.

Retinal microvascular function was assessed by vessel diameters through central retinal venular equivalent (CRVE) and central retinal arteriolar equivalent (CRAE) based on the improved Knudtson-Hubbard formula. Measurements were based on the six largest vessels within 0.5–1.0 disc diameter from the optic margin. Retinal arteriolar and venular vasodilation was assessed by measuring the mean diameter change over a 40-second flicker-light stimulus relative to baseline [6]. 

Skin microvascular function was assessed as flowmotion (oscillations) and heat-induced skin hyperemia. Flowmotion was obtained by laser-Doppler flowmetry, primarily measuring arterioles and venules at the dorsal wrist for 25 min. Power density signals (arbitrary perfusion units, PU [2]) were derived via fast-Fourier transformation of frequency domains, reflecting endothelial (0.01–0.02 Hz), neurogenic (0.02–0.06 Hz), myogenic (0.06–0.15 Hz), respiratory (0.15–0.40 Hz), and cardiac (0.40–1.60 Hz) activity. Heat-induced hyperemic skin responses were also assessed using laser-Doppler flowmetry; perfusion was recorded during 2 min baseline and during 23 min of heating at 44 degrees Celsius. Hyperemic response was expressed as a percentage change from average baseline to heating perfusion units.

Renal microvascular function was assessed using the urinary albumin-to-creatinine ratio (UACR), a marker of renal endothelial integrity, by dividing albumin and creatinine levels from spot urine samples.

### Covariates

Clinical data of patients with HFpEF were collected during the most recent hospital visit and included demographics, medical history, cardiovascular risk factors (such as smoking: never, former, current), vital signs, symptoms, medication use, cardiac diagnostic results (such as echocardiography and right heart catheterisation), and routine blood tests. Clinical data were used up to 6 months before inclusion using a standardised infrastructure to ensure high-quality data collection and processing [11]. 

The same clinical data were prospectively obtained in control individuals from The Maastricht Study through standardised questionnaires, examinations, and additional diagnostics by trained staff, as described previously [9]. 

In both groups, brachial blood pressure was assessed in a supine resting state every 5 min for at least 20 min (Accutorr Plus device, Datascope Inc.) and averaged. Physical activity was assessed using a modified Champs Activities Questionnaire for Older Adults [9]. Total activity was calculated as the sum of the weekly duration (midpoint) of all reported activities. Moderate-to-vigorous intensity physical activity (MVPA) was calculated similarly from all activities labelled moderate-to-vigorous. An independent ophthalmologist reviewed all fundus images to identify confounding ocular conditions, such as retinopathy or glaucoma.

### Statistical analysis

A statistical plan was created prior execution of the study. Baseline characteristics are presented as absolute numbers (n) and percentages (%) for categorical variables, and as mean with standard deviation (SD) or median with interquartile ranges (IQR) for continuous variables, depending on their distribution. Clinical differences between patients with HFpEF and control individuals, and between sexes, were assessed using independent T-test or Mann-Whitney U test for continuous variables, depending on the data distribution. Categorical data was evaluated using Chi-square or Fisher exact test, depending on minimum expected counts per category.

To answer the primary research questions, linear regression models were constructed in a multiple imputed dataset (Supplemental Methods) to assess the difference of the dependent variables (results of microvascular assessments) between patients with HFpEF and control individuals (independent variable) reporting absolute differences (β) with corresponding 95% confidence intervals (95%CI). Each marker of microvascular function was tested in separate analyses. We constructed *model 1* (crude model), *model 2* (adjusted for age and sex), *model 3* (model 2 + systolic blood pressure, body mass index, DM status, and haemoglobin), and *model 4* (final model: model 3 + smoking status). Due to female predominance in HFpEF and abundance of a metabolic HFpEF phenotype (mainly driven by DM) [5] and oversampling of DM within the control group, all final model analyses were exploratively tested for interactions with sex and DM status, and stratified in case a pooled p_interaction_ < 0.10. Continuous variables not meeting linear regression assumptions were log-transformed, are reported as such, and all met the assumptions thereafter.

Sensitivity analyses were performed to evaluate the robustness of the linear regression results (detailed in Supplemental Methods). Analyses were performed using SPSS (version 28). Case-control matching and corresponding analyses were performed with RStudio (version 2023.03). A two-sided *p*-value of < 0.05 was considered statistically significant.

## Results

### Clinical characteristics

This study included 138 patients with HFpEF and 2140 control individuals (Fig. S1). Patients with HFpEF were older, more frequently female, less physically active, had more often hypertension and obesity, had lower average blood pressures, higher heart rates, and more frequently used cardiovascular medications (Table 1, Supplemental Fig. S2). In laboratory analyses, HFpEF patients had lower haemoglobin levels, higher leucocyte count, lower eGFR, more often albuminuria, and elevated NT-proBNP levels. Echocardiography showed expected findings between patients versus controls: a similar LV ejection fraction and more signs of elevated filling pressures, hypertrophy, and diastolic dysfunction markers in HFpEF.

**Table 1:** Clinical characteristics in patients with HFpEF and control individuals

Clinical variables	*n*	Patients with HFpEF (*n* = 138)	Control individuals (*n* = 2140	*p*-value
Age (years)	138/2140	75 ± 6	66 ± 4	< 0.001
Female sex	138/2140	95 (69%)	975 (46%)	< 0.001
Postmenopausal	95/964	95 (100%)	962 (99.8%)	0.999
Hormone replacement therapy	81/975	0 (0%)	1 (0.1%)	0.999
Physical activity				
Self-reported moderate-vigorous physical activity, hours/week	137/1916	0.8 [0–3.0]	4.5 [1.8–7.8]	< 0.001
Self-reported total physical activity, hours/week	137/1916	9.5 [5.8–14.5]	12.8 [7.8–19.0]	< 0.001
Comorbidities				
DM	138/2140	31 (23%)	624 (29%)	0.092
Hypertension	138/2139	112 (81%)	1390 (65%)	< 0.001
Coronary artery disease	138/2140	17 (12%)	220 (10%)	0.447
Obesity	138/2138	50 (36%)	448 (21%)	< 0.001
Smoker, current	138/2127	7 (5%)	214 (10%)	0.056
Smoking pack years	104/1701	1.5 [0–20]	2.0 [0–17.1]	0.684
Retinopathy in either eyes	132/2093	3 (2%)	44 (2%)	0.756
Physical examination				
Systolic BP (mmHg)	125/2008	127 ± 17	130 ± 14	0.057
Diastolic BP(mmHg)	125/2008	71 ± 10	76 ± 7	< 0.001
Heart rate (bpm)	138/2134	72 ± 13	68 ± 11	0.003
Body mass index (BMI) (kg/m^2^)	138/2138	29.6 ± 5.9	27.1 ± 4.3	< 0.001
Medications				
Lipid-modifying medications	138/2140	77 (56%)	879 (41%)	< 0.001
Anti-hypertensive medications	138/2140	127 (92%)	997 (47%)	< 0.001
Respiratory medication for COPD or asthma	138/2140	33 (24%)	169 (8%)	< 0.001
Diabetes medication	138/2140	25 (18%)	448 (21%)	0.429
Anticoagulants	138/2140	115 (83%)	512 (24%)	< 0.001
Laboratory findings				
Hemoglobin (mmol/l)	125/2114	8.2 ± 0.8	8.8 ± 0.7	< 0.001
Mean corpuscular volume (fl.)	121/2113	92 ± 5	91 ± 4	0.004
Leucocytes (10^9^/L)	121/1978	7.3 [5.7–8.7]	5.5 [4.7–6.6]	< 0.001
HbA1c (%)	114/2139	39.5 [35–46]	38 [35–44]	0.171
Albuminuria (Urinary albumin-to-creatinine ratio ≥ 3 g/mol)	93/1121	25 (27%)	53 (5%)	< 0.001
Estimated glomerular filtration (e-GFR) CKD-EPI (L/min/1.73 m)	124/2138	55.4 [42.1–69.5]	78 [69–88]	< 0.001
Total-to-HDL cholesterol (ratio)	117/2140	3.2 [2.8–4.1]	3.4 [2.8–4.2]	0.228
NT-proBNP (pg/dL)	117/875	526.9 [230.5–955.6]	59.7 [35.4–100.4]	< 0.001
NT-proBNP > 125 pg/dL	117/875	109 (93%)	147 (17%)	< 0.001
Echocardiography				
LV ejection fraction	138/307	60 ± 5	60 ± 3	0.251
E wave (cm/s)	128/306	89 ± 26	65 ± 14	< 0.001
A wave (cm/s)	84/301	77 ± 24	73 ± 16	0.193
E/A ratio	84/301	1.0 [0.8–1.5]	0.9 [0.8–1.0]	0.001
Septal E’	128/286	7.0 ± 2.2	7.0 ± 1.7	0.922
Lateral E’	128/288	9.2 ± 2.9	8.9 ± 2.1	0.366
Average E/E’	123/286	11.5 ± 4.0	8.6 ± 2.2	< 0.001
LV mass index (gr./m^2^)	136/303	74 ± 19	68 ± 14	< 0.001
Relative wall thickness	136/303	0.38 ± 0.06	0.36 ± 0.06	0.017
Left atrial volume index (ml/m^2^)	129/298	46 ± 15	30 ± 7	< 0.001
TAPSE (mm)	64/69	21 ± 5	22 ± 4	0.343
Peak tricuspid regurgitation (m/s)	121/303	2.5 [2.3–2.8]	2.0 [1.7–2.3]	< 0.001

Data presented as mean ± standard deviation, median [inter-quartile ranges], or count (percentage). CKD-EPI, Chronic Kidney Disease Epidemiology Collaboration equation; COPD, chronic obstructive pulmonary disease; DM, diabetes mellitus; HbA1c, hemoglobine A1c; HDL, high-density-lipoprotein; HFpEF, heart failure with preserved ejection fraction; LV, left ventricle; NT-proBNP, N-terminal pro–B-type natriuretic peptide; TAPSE, tricuspid annular plane systolic excursion

### Retinal microvascular measurements

Retinal venular and arteriolar diameter measurements (CRVE and CRAE, respectively) were available in 131 (95%) patients and 2030 (95%) controls (Table 2). Retinal CRVE were narrower in HFpEF patients compared to controls in both crude (*p* < 0.001) and confounder-adjusted models (*p* = 0.008) (Table 3). CRAE were narrower in patients versus controls in crude analysis (*p* = 0.001), but not after confounder adjustments (*p* = 0.073).

**Table 2:** Microvascular characteristics in patients with HFpEF and control individuals

Microvascular assessments	*n*	HFpEF patients (*n* = 138)	Controls (*n* = 2140)
Retinal microvasculature			
Retinal calibers			
Central retinal arteriolar equivalent (CRAE) caliber, µm	133/2030	130 ± 19	136 ± 19
Central retinal venular equivalent (CRVE) caliber, µm	133/2030	194 ± 28	208 ± 30
Flicker-light induced vasodilation			
Arteriolar dilation, %	103/2140	1.2 [1.2–3.3]	2.0 [0.5–4.2]
Venular dilation, %	110/2102	3.2 [1.9–4.5]	3.4 [2.2–5.0]
Skin microvasculature			
Vasomotion			
Endothelial power, PU^2^	131/1705	35,746 [12,930 − 100,767]	44,314 [15,812 − 124,219]
Myogenic power, PU^2^	131/1705	5,453 [2,095 − 20,640]	4,540 [1,555 − 12,982]
Neurogenic power, PU^2^	131/1705	16,412 [7,562 − 57,146]	20,302 [7,648 − 56,333]
Respiratory power, PU^2^	131/1705	8,673 [922–8,617]	1,514 [529–4,866]
Cardiac power, PU^2^	131/1705	1,712 [722–6,611]	1,506 [580–4,250]
Total power, PU^2^	131/1705	68,238 [26,352 − 200,864]	76,733 [30,009–206,974]
Heat-induced hyperemia			
Heat-induced hyperemic response, %	131/1630	1153 [693–1670]	1031 [611–1568]
Renal microvasculature			
Urinary albumin-to-creatinine ratio (UACR), g/mol	93/1121	1.2 [0.6–3.6]	0.3 [0.2–0.6]
Albuminuria (UACR ≥ 3 g/mol)	93/1121	25 (27%)	53 (5%)

Results from difference testing between groups are reported in Tables 3 and 4. Abbreviations as in Table 1. PU, (arbitrary) perfusion units.

**Table 3:** Linear regression models for HFpEF status on retinal microvascular assessments

Variable	Β	95%CI	SE β	Standardized β*	*p*-value
CRVE, µm					
Model 1	− 13.6	− 18.9– − 8.4	2.7	− 0.096	< 0.001
Model 2	− 7.1	− 12.9– − 1.3	3.0	− 0.049	0.017
Model 3	− 8.2	− 14.2– − 2.2	3.1	− 0.056	0.008
Model 4 (final)	− 8.1	− 14.1– − 2.1	3.1	− 0.055	0.008
Interaction between HFpEF status and sex					*0.023*
Stratified: males	1.2	− 8.9–11.4	5.2	0.009	0.812
Stratified: females	− 13.8	− 21.4– − 6.2	3.9	− 0.119	< 0.001
Interaction between HFpEF status and DM					*0.426*
CRAE, µm					
Model 1	− 5.9	− 9.2– − 2.5	1.7	− 0.071	0.001
Model 2	− 1.9	− 5.6–1.8	1.9	− 0.028	0.317
Model 3	− 3.4	− 7.2–0.4	1.9	− 0.045	0.082
Model 4 (final)	− 3.5	− 7.3–0.3	1.9	− 0.045	0.073
Interaction between HFpEF status and sex					*0.466*
Interaction between HFpEF status and DM					*0.847*
Retinal venular dilatation, %					
Model 1	− 0.23	− 0.69–0.24	0.24	− 0.014	0.341
Model 2	− 0.31	− 0.82–0.20	0.26	− 0.024	0.238
Model 3	− 0.24	− 0.77–0.28	0.27	− 0.020	0.368
Model 4 (final)	− 0.23	− 0.75–0.30	0.27	− 0.019	0.392
Interaction between HFpEF status and sex					*0.938*
Interaction between HFpEF status and DM					*0.606*
Retinal arteriolar dilatation, %					
Model 1	− 0.65	− 1.25– − 0.048	0.31	− 0.041	0.034
Model 2	− 0.19	− 0.85–0.47	0.34	− 0.015	0.571
Model 3	− 0.20	− 0.87–0.48	0.35	− 0.016	0.569
Model 4 (final)	− 0.18	− 0.86–0.49	0.35	− 0.015	0.593
Interaction between HFpEF status and sex					*0.683*
Interaction between HFpEF status and DM					*0.906*

*of original data model. Abbreviations as in Table 2. Model 1: crude model with HFpEF status. Model 2: addition of age and sex. Model 3: addition of diabetes mellitus status, systolic blood pressure, body mass index, and Haemoglobin. Model 4: addition of smoking status. Results from model 5 (model 4 with addition of hours/week moderate to vigorous physical activity) are summarised in Supplemental Table S11. Italic *p*-values represent p_interaction_

**Fig. 1:**
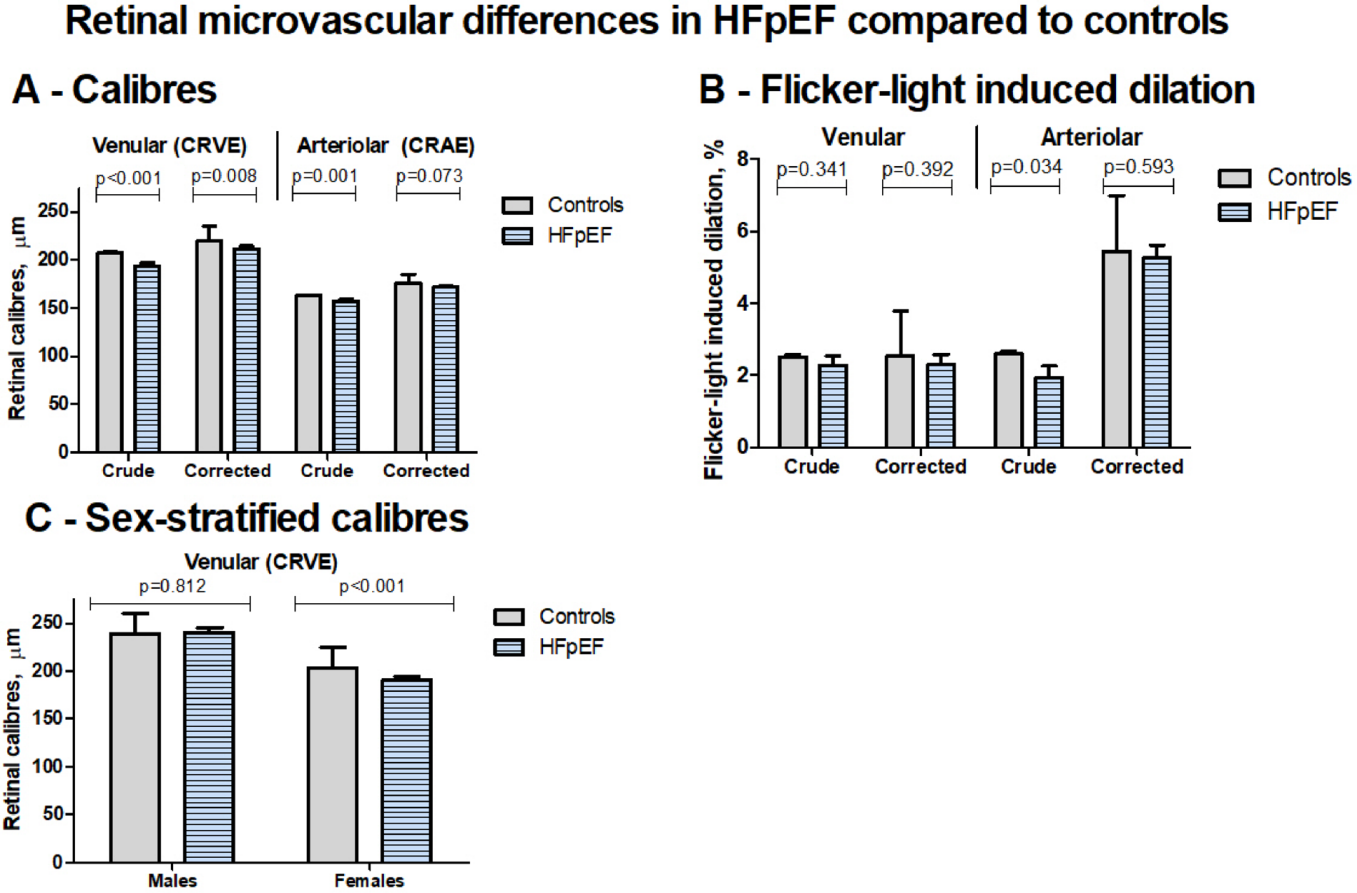
Microvascular retinal differences between patients with HFpEF and control individuals. Panel A shows the retinal dilation in response to flicker-light by arterioles and venules. Values for controls are based on the intercept for each respective linear regression model. Values for HFpEF patients represent the mean difference compared to control individuals. Each blunt head represents the standard error of measurement. Conclusions are made based on the corrected results of model 4 (adjustment for age, sex, systolic blood pressure, body mass index, diabetes mellitus status, haemoglobin, and smoking status) and are displayed here as Corrected. Differences in arteriolar and venular retinal diameters (CRAE and CRVE, respectively) are shown in panel B. Sex-specific CRVE differences are shown in panel C.

Flicker-light-induced retinal venular dilation was similar, and arteriolar dilation was reduced in HFpEF patients compared to controls in crude analyses (*p* = 0.341 and 0.034, respectively; Fig. 1; Table 2). However, no significant differences were observed after confounder-adjustment for either venular or arteriolar dilatation (*p* = 0.392 and 0.593, respectively) (Table 3).

### Skin microvascular measurements

**Fig. 2:**
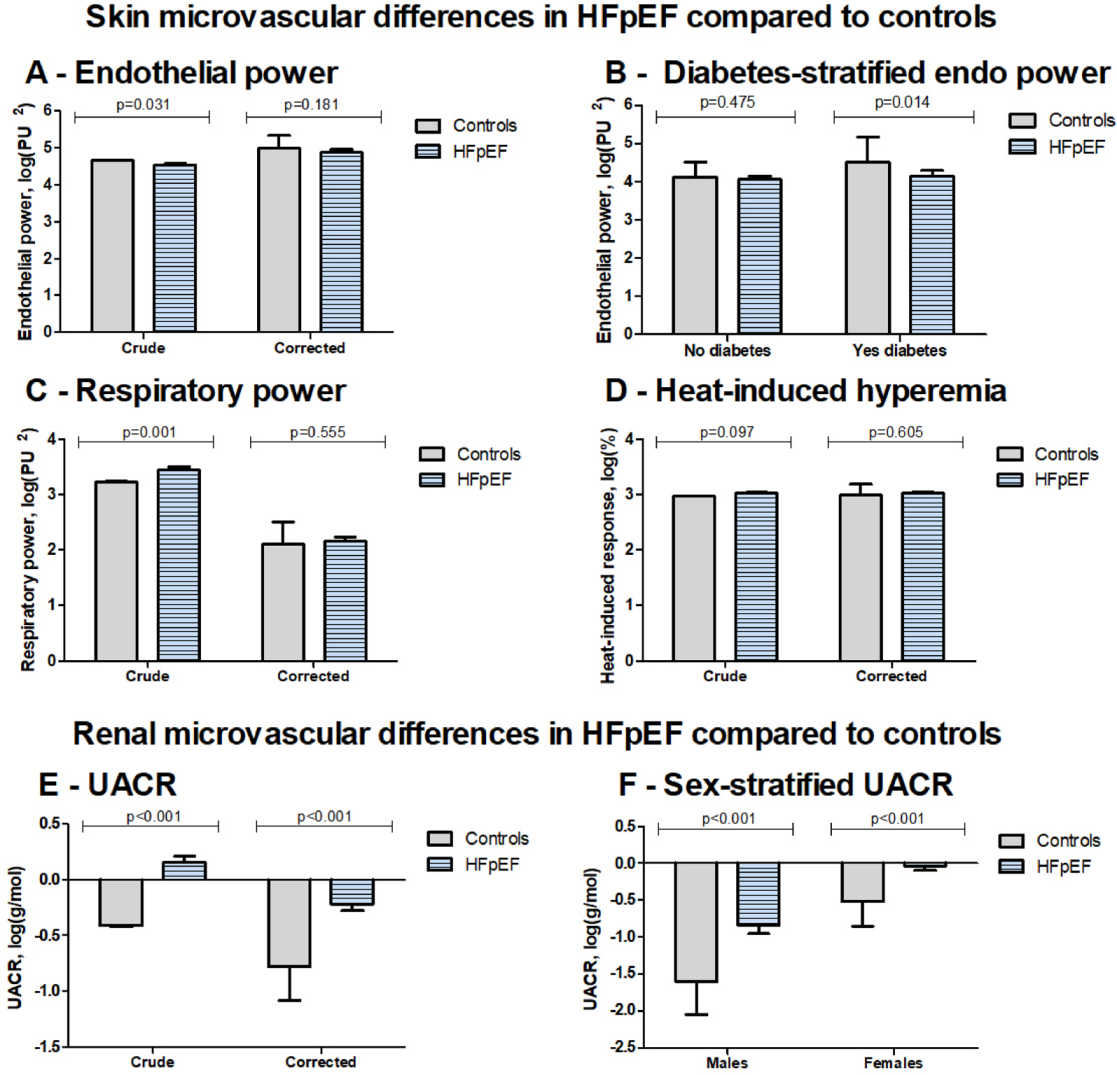
Microvascular skin and renal differences between patients with HFpEF and control individuals. Results are displayed as in Fig. 2, and interpretation should focus on differences from confounder-corrected analyses. Panel A shows the endothelial power based on flowmotion signals without adjustment and with full adjustment (Corrected). Because of an interaction with diabetes mellitus, panel B shows endothelial power differences between patients with HFpEF and control individuals stratified for diabetes mellitus status. Group differences in respiratory power based on flowmotion signals are depicted in panel C. Panel D displays the differences in heat-induced skin hyperemia in patients with HFpEF and control individuals. Panel E shows the difference in UACR between groups without adjustment and with full adjustments (Corrected). Panel F shows the fully adjusted UACR group differences stratified by sex

Flowmotion analyses of skin microvascular perfusion showed lower endothelial power and higher respiratory power in patients with HFpEF versus controls in crude analyses (*p* = 0.031 and 0.001, respectively; Fig. 2). In confounder-adjusted models, however, both power signals were not significantly different between patients and control individuals (*p* = 0.181 and 0.555, respectively; Table 4).

**Table 4:** Linear regression models for HFpEF status on skin and renal microvascular assessments

Variable	Β	95%CI	SE β	Standardized β*	*p*-value
Endothelial power, PU^2^ (log-transformed)					
Model 1	− 0.13	− 0.25– − 0.01	0.06	− 0.044	0.031
Model 2	− 0.13	− 0.26– − 0.002	0.07	− 0.043	0.050
Model 3	− 0.09	− 0.22–0.05	0.07	− 0.026	0.210
Model 4 (final)	− 0.09	− 0.23–0.05	0.07	− 0.027	0.181
Interaction between HFpEF status and sex					*0.962*
Interaction between HFpEF status and DM					*0.048*
Stratified: no DM	− 0.06	− 0.22–0.10	0.08	− 0.017	0.475
Stratified: yes DM	− 0.36	− 0.65– − 0.07	0.15	− 0.111	0.014
Respiratory power, PU^2^ (log-transformed)					
Model 1	0.21	0.08–0.34	0.07	0.075	0.001
Model 2	0.0002	− 0.15–0.15	0.07	0.007	0.998
Model 3	0.05	− 0.10–0.20	0.08	0.023	0.495
Model 4 (final)	0.05	− 0.11–0.20	0.08	0.022	0.555
Interaction between HFpEF status and sex					*0.228*
Interaction between HFpEF status and DM					*0.149*
Heat-induced hyperemia response, % (log-transformed)					
Model 1	0.05	− 0.01–0.11	0.03	0.036	0.097
Model 2	0.03	− 0.04–0.09	0.03	0.020	0.424
Model 3	0.01	− 0.05–0.08	0.03	0.014	0.422
Model 4 (final)	0.02	− 0.05–0.08	0.03	0.016	0.517
Interaction between HFpEF status and sex					0.166
Interaction between HFpEF status and DM					0.512
Urinary albumin-to-creatinine ratio (UACR), g/mol (log-transformed)					
Model 1	0.57	0.47–0.66	0.05	0.288	< 0.001
Model 2	0.54	0.43–0.65	0.05	0.271	< 0.001
Model 3	0.56	0.45–0.67	0.06	0.286	< 0.001
Model 4 (final)	0.56	0.45–0.67	0.06	0.285	< 0.001
Interaction between HFpEF status and sex					*0.002*
Stratified: males	0.76	0.55–0.96	0.11	0.278	< 0.001
Stratified: females	0.48	0.36–0.60	0.06	0.331	< 0.001
Interaction between HFpEF status and DM					*0.883*

Results of all flowmotion components are summarised in Supplemental Table S1. Results from model 5 (model 4 with addition of hours/week moderate to vigorous physical activity) are summarised in Supplemental Table S11. Abbreviations as in Table 3. Italic *p*-values represent p_interaction_

Heat-induced skin hyperemia was not different in HFpEF patients compared to controls (crude *p* = 0.097, confounder-adjusted model *p* = 0.605; Table 4).

### Renal microvascular measurements

Patients with HFpEF had higher UACR than controls (*p* < 0.001). This difference remained present after adjustments for all pre-defined confounders (*p* < 0.001; Fig. 2), including baseline eGFR (*p* < 0.001) (Supplemental Table S2; Table 4).

### Sex differences in microvascular measurements

In confounder-adjusted models, sex significantly interacted with HFpEF status for both CRVE and UACR (p_interaction_=0.023 and 0.002, respectively, Tables 3 and 4). Stratified analyses revealed lower CRVE in females with HFpEF compared to female controls (beta − 13.0 μm [95%CI − 24.2 to − 1.8], *p* = 0.023), but this difference was not observed in males (*p* = 0.801) (Fig. 1). For UACR, male patients had relatively higher UACR versus controls (beta 0.76 g/mol(log-transformed) [95%CI 0.55–0.96], *p* < 0.001) compared to female patients versus control individuals (beta 0.48 g/mol(log-transformed) [95%CI 0.36–0.60], *p* < 0.001) (Fig. 2). In crude comparisons between male and female HFpEF patients, CRVE, respiratory power, and UACR tended to lower values in females (Supplemental Table S3).

Regardless of HFpEF status, females sex was independently associated with CRAE and CRVE widening, more retinal venular and arteriolar dilatation, lower endothelial and respiratory power, larger heat-induced hyperemia response, and lower UACR (Supplemental Tables S4–11).

### Diabetes status differences in microvascular measurements

In the confounder-adjusted models, DM status interacted with skin endothelial power (p_interaction_=0.002). Stratified analyses revealed lower endothelial power in patients with HFpEF and DM compared to controls with DM (beta − 0.36PU^2^ (log-transformed) [95%CI − 0.65–0.07), *p* = 0.014; Table 4, Supplemental Table S1). Higher BMI was also independently associated with lower endothelial power, whether DM was present or not.

Regardless of HFpEF status, DM was independently associated with narrower CRAE, lower retinal venular and arteriolar dilatation, smaller heat-induced hyperemia response, and higher UACR (Supplemental Tables S4–11).

### Additional analyses

Additional analyses confirmed the robustness of the main findings. HFpEF patients (*n* = 102) matched to control individuals (*n* = 102 and *n* = 204), based on age, sex, DM, hypertension, and body mass index, revealed similar results to the primary linear regression analyses (Table 5, Supplemental Table S16).

**Table 5:** Clinical and microvascular characteristics from sensitivity analyses in patients with HFpEF and matched controls

	HFpEF patients (*n* = 102)	Controls (*n* = 102)	*p*-value
Clinical characteristics			
Age, years	73 (69, 75)	73 (69, 75)	0.415
Female sex, n (%)	69 (68)	67 (66)	0.882
Diabetes mellitus, n (%)	26 (25)	24 (24)	0.871
Hypertension, n (%)	80 (78)	81 (79)	1
Body mass index, kg/m^2^	28.9 (26.2, 33.7)	29.4 (26.9, 32.7)	0.556
Retinal microvasculature			
Retinal calibres			
CRVE, µm	197.33 ± 25.27	207.54 ± 32.19	0.015
CRAE, µm	131.61 ± 18.92	135.93 ± 19.87	0.124
Flicker-light induced vasodilation			
Arteriolar dilation, %	1.41 (0.01, 3.56)	1.26 (0.39, 3.21)	0.88
Venular dilation, %	3.01 (1.92, 4.28)	3.66 (2.31, 4.89)	0.06
Skin microvasculature			
Vasomotion			
Cardiac power, PU^2^	1511 (728, 6076)	1559 (662, 3645)	0.636
Endothelial power, PU^2^	37,558 (12080, 74805)	30,457 (11080, 96395)	0.99
Myogenic power, PU^2^	5335 (2136, 16061)	4082 (1213, 9271)	0.156
Neurogenic power, PU^2^	16,356 (8022, 37647)	17,946 (7006, 45562)	0.965
Respiratory power, PU^2^	2515 (905, 6955)	1503 (452, 4523)	0.09
Total power, PU^2^	66,617 (26092, 166485)	58,831 (24312, 167812)	0.732
Heat-induced hyperemia			
Heat-induced hyperemia response, %	1230 (795, 1663)	1115 (701, 1767)	0.476
Renal microvasculature			
Urinary albumin-to-creatinine ratio (UACR), g/mol	1.3 (0.6, 4.1)	0.3 (0.2, 0.5)	< 0.001

Data presented as mean ± standard deviation, median (inter-quartile ranges), or count (percentage). Data distribution of age, CRVE and UACR before and after matching is displayed in Supplemental Fig. S3. CRAE, central retinal arteriolar equivalent; CRVE, Central retinal venular equivalent; HFpEF, heart failure with preserved ejection fraction; PU, arbitrary perfusion unit

Sensitivity analyses consistently confirmed the qualitative microvascular differences observed in the primary linear regression analyses between HFpEF patients and controls after confounder-adjustment (model 4).

Adjusting for physical activity, replacing DM status with HbA1c, and complete-case analyses yielded similar results, including the observed interactions (Supplemental Tables S11–13). Linear regression with UACR based on 24-hour urine collection instead of urine portion of control individuals increased sample size (*n* = 2135 instead of 1121) and yielded similar results (final model beta 0.34 [95%CI 0.22–0.47], *p* < 0.001). Excluding outliers did not alter observed associations (data not shown). No relevant multicollinearity was identified.

Logistic regression corrected for confounders, same as model 4, showed UACR as most potent MVD marker associated with HFpEF in both sexes, while narrower CRVE was also strongly associated with HFpEF in females (Supplemental Table S14). Analysing a subset of patients in which NT-proBNP was available (*n* = 117 HFpEF and *n* = 875 controls) and added to the new model 5c, blunted the association between UACR and HFpEF status in females (*p* = 0.669) but not in males (*p* = 0.026), while narrower CRVE remained associated with HFpEF in females (*p* = 0.005) (Supplemental Table S15).

## Discussion

The present study, the largest case-control cohort on MVD and HFpEF to date, provides new evidence on systemic MVD in HFpEF. We demonstrate that patients with HFpEF show microvascular differences in all microvascular beds studied compared to control individuals in crude analyses. However, after confounder adjustments, these differences were most prominently in the retina and kidney. Retinal venular diameter (CRVE) narrowing was mainly present in females with HFpEF, while no notable difference in retinal response to flicker-light was observed. Measures reflecting renal MVD (UACR) were higher in HFpEF patients than controls, with a more pronounced difference in male than female patients. Skin microvascular differences between patients with HFpEF and control individuals were only observed in participants with concomitant DM. The present study’s findings support that MVD is likely a more systemic process than only coronary in HFpEF [1, 3], while also revealing subgroup-specific microvascular differences.

### Retinal microvascular dysfunction in HFpEF

We observed remarkable retinal diameter differences between HFpEF patients and control individuals. Our finding of retinal arteriolar narrowing in HFpEF aligns with previous associations with cardiovascular disease (Supplemental Table S17) [12–15]. However, the substantial retinal venular narrowing in HFpEF contrasts earlier general population studies linking venular widening with LV remodelling– which can be a precursor of HFpEF development– and incident HF, especially in females [12–14], although one population study reported venular narrowing to be associated with incident HT [15]. The observed directionally opposite association of venular narrowing rather than widening in HFpEF was clearly more than expected based on increasing age [16]. The venular narrowing possibly reflects specific combined retinal neuronal and vascular degeneration in HFpEF [17, 18]. Moreover, coronary microvascular dysfunction, possibly sharing pathophysiology with HFpEF [1], has been noted to present smaller coronary microvasculature and higher resting flow in females compared to males [19]. These findings may relate to the observed retinal vessel narrowing, although a direct comparison between the two vascular beds in HFpEF is essential for validation. Thus, retinal venular narrowing, especially in females, may either reflect a consequence of HFpEF or signify a unique venular adaption during progression towards HFpEF that is absent in other HF phenotypes. The specific alterations of the venules may be particularly relevant because they are the main site for leukocyte extravasation and inflammatory response, which may be affected by smaller vessel diameters [20]. Given the contrasting results of longitudinal studies and incident HF, further investigations are warranted to confirm these speculations and elucidate the consequences for cardiac pathophysiology.

A blunted retinal arteriolar dilation response to flicker-light in HFpEF may be expected given its dependency on nitric oxide (NO) [10], as decreased systemic NO bioavailability and consequential endothelial-myocyte oxidative stress is postulated as one of the primary mechanisms for cardiac remodelling in HFpEF [4]. Such results, however, were not observed when results were confounder-adjusted. Earlier studies, unadjusted for confounders, reported reduced retinal arteriolar but not venular response to flicker-light in patients with cardiovascular risk factors and further reduction in patients with HF [21]. Similar to patients with cardiovascular risk factors, a large population-based study reported reduced arteriolar dilation response (2.3%) in patients with DM after confounder adjustments [22]. However, our study reports considerably lower arteriolar dilatation in both control individuals and patients with HFpEF (1:1 matched dataset showed 1.5% and 1.4%, respectively) compared to previous findings [21, 22], likely attributable to differing flicker-light protocols, confounder adjustments and variations in study populations. Specifically, our HF population included only HFpEF instead of mainly HFrEF [21] or no HF at all [22]included almost double amount of females, and our patients were more than a decade older compared to prior work [21, 22]. Although hyperglycaemia has been reported to be the primary driver of retinal microvascular dysfunction in younger individuals and low-grade inflammation did not contribute to this association [23], it remains to be elucidated which pathomechanism other than increasing age would drive the progressive decrease in retinal dilation in both patients with HFpEF and control individuals with prevalent cardiovascular risk factors. Likely, these mechanisms include underlying impaired neurovascular coupling due to cardiovascular risk factors such as hyperglycaemia, which we also observed as independently associated with lower retinal dilatation regardless of HFpEF, and inflammation [4, 10]. 

### Skin and renal microvascular dysfunction in HFpEF

An expected decreased heat-induced response [22], attributable to lower NO availability or endothelium-derived hyperpolarising factors [1], was not observed in HFpEF. Smaller studies have suggested altered skin hyperemia in HFpEF using alternate pharmacological stimuli that may more specifically target underlying mechanisms [1, 24], albeit with limited generalisability due to insufficient confounder adjustments or a low number of HFpEF cases. In the present study, patients with HFpEF had decreased endothelial power of skin flowmotion compared to control individuals only when concomitant DM was present. These findings are in line with previously reported data from The Maastricht Study [25]. Taking these data together, it is likely that multiple underlying processes, like hyperglycaemia and obesity-induced inflammation, lead to skin MVD in the context of HFpEF.

Increased UACR and albuminuria in patients with HFpEF compared to controls, even after additional adjustment for renal function, echoes previous studies showing associations between higher UACR/albuminuria and adverse cardiac remodelling in patients with HFpEF or those at risk for HFpEF [26, 27]. Additionally, albuminuria has been associated with decreased echocardiographic coronary flow reserve and adverse outcomes in HFpEF [26, 28]. Two mechanisms likely contribute to these findings. First, albuminuria reflects glomerular microvascular damage and is suggested as a marker of systemic MVD [29]. Second, venous congestion in HF can cause albuminuria [30]. Furthermore, the pronounced sex disparity in UACR between patients and controls aligns with findings from a population study in which more albuminuria was reported in male versus female patients with DM [31]. In HFpEF, such differences may relate to sex-varied renal vascular resistance [30] and the renin-angiotensin-aldosterone system [32], although more sex-specific physiological differences are to be elucidated. Finally, elevated UACR may be an effect-modifier for several therapies with renal implications [33], such as mineral corticosteroid antagonists or sodium-glucose transporter-2 inhibitors, urging further mechanistic and longitudinal studies with UACR to optimise treatment efficiency in HFpEF.

### Clinical implications and future perspectives

The present study underscores associations of MVD across different microvascular beds with HFpEF, gradually building towards delineating systemic MVD as potential contributor of HFpEF development. Presence of systemic MVD holds promise for HFpEF screening, risk-stratification, and targeted therapies [1]. Achieving these milestones will require future work exploring temporal sequences, and therapy impacts on direct microvascular measures. The lack of observed differences in responsive retinal dilatation and skin heat-induced hyperemia between patients with HFpEF and control individuals hints at other pathomechanisms than only NO-dependent vasodilation, such as hyperglycaemia, increased sympathetic nervous activity, or altered rheology [1, 34, 35]. These specific mechanisms may depend on sex, comorbidities, and phenotypes, as depicted by our reported sex-dependent retinal and renal MVD, and DM-dependent microvascular skin differences. In line herewith, a recent study showed mainly decreased skin hyperemic response to insulin instead of (NO-dependent) acetylcholine or nitroprusside in patients with DM and signs of HFpEF [24], which improved by empagliflozin treatment in a recent study [36]. Likely, a combination of underlying processes co-occurs in HFpEF. Therefore, carefully selecting HFpEF subgroups may be crucial for studies aiming to dissect and target specific underlying pathomechanisms. Phenogrouping efforts [37], when integrated with microvascular assessments, may facilitate these advances. Moreover, how direct microvascular measures relate to cardiac MVD or patient’s functional status is largely unknown. Several studies are ongoing to answer parts of these knowledge gaps (NCT06046612, NCT05610410). Still, more longitudinal studies are warranted to tackle the causal complexity between systemic MVD and HFpEF and to explore its sex-specificity.

### Strengths and limitations

A key strength of our study is its comprehensiveness, including the largest case-control cohort on systemic MVD and HFpEF to date. Additionally, patients with HFpEF were extensively screened by an expert centre, while control individuals were assessed similarly by a population-based study in the same region. Uniform equipment and protocols were employed for all microvascular assessments in both groups, enhancing the internal validity of the findings. Oversampling of control individuals, including those with DM, allowed for comprehensive confounder adjustments. We expect that oversampling of DM within the control group led to worse microvascular function compared to healthy control individuals [22]. Therefore, our study is more robust to identify HFpEF-specific findings, given the smaller expected contrast between cases and controls. The results were obtained by several direct and independent microvascular assessments in different vascular beds, including renal biomarkers, and were consistent among multiple sensitivity analyses.

Several factors limited the study. First, most control individuals were younger than the HFpEF patients, introducing potential residual confounding for reduced microvascular function [16] and limiting the number of matches between both groups. Moreover, patients with HFpEF were more often females, warranting further studies extrapolating the results of male HFpEF patients and for more definitive sex-specific conclusions. Second, the study’s cross-sectional design precludes discerning whether observed microvascular differences in patients with HFpEF were a driver for HFpEF or a consequence of the syndrome. Third, although care was taken to ensure adequate confounder adjustment, differences may have been driven by residual confounders not included in the analyses (such as oestrogen levels and receptor activity [1, 38]). Models 5-5c were likely overadjusted due to the inclusion of possible mediators, but the results remained qualitatively similar to the final model 4. Fourth, extensive echocardiography analyses were available only in a subset of first-enrolled control individuals (14%) and could have led to the inclusion of patients with HFpEF without apparent symptoms in the control group. However, this group is small, as control individuals were excluded based on symptoms, impaired systolic LV function or relevant valvular heart disease based on focused echocardiography analyses, and self-reported HF within 1 year after enrolment. Fifth, the regional confinement of our study to the southern part of the Netherlands, with a clear Caucasian predominance, necessitates validation in different ethnic groups.

## Conclusions

Patients with HFpEF show microvascular differences across all vascular beds studied compared to controls. However, after adjustment for important confounders, these differences remained significant in eyes and kidneys. The findings across multiple organs support that MVD is likely a more systemic process than only coronary MVD in HFpEF. Moreover, our findings support the general notion that the underlying pathophysiology in HFpEF may be sex-specific. Thus, future studies targeting MVD in HFpEF may need sex-specific approaches.

Figure created in BioRender (i88u523).

## Electronic supplementary material

Below is the link to the electronic supplementary material.

Supplementary Material 1.

## Data Availability

No datasets were generated or analysed during the current study.

## Abbreviations

BMI: Body mass index
CRAE: Central retinal arteriolar equivalent
CRVE: Central retinal venular equivalent
eGFR: Estimated glomerular filtration rate
HbA1c: Haemoglobin A1c
HFpEF: Heart failure with preserved ejection fraction
LV: Left ventricle
MVD: Microvascular dysfunction
MVPA: Moderate-to-vigorous intensity physical activity
NO: Nitric oxide
NT-proBNP: N-terminal pro-B-type natriuretic peptide
OR: Odds ratio
PROSE-HFpEF: Prospective Case-Control Study to Evaluate Systemic Microvascular Dysfunction in Heart Failure with Preserved Ejection Fraction in Males and Females
PU: (arbitrary) Perfusion units
UACR: Urinary albumin-to-creatinin ratio
