# Supplementary material for: Microvascular dysfunction across organs in heart failure with preserved ejection fraction: the PROSE-HFpEF case-control study
Source: Cardiovasc Diabetol. 2025 Jul 30;24:310. doi: 10.1186/s12933-025-02850-1 (PMC12312529; doi:10.1186/s12933-025-02850-1)
Supplement: Supplementary file 1 — Supplementary Material 1. [file 12933_2025_2850_MOESM1_ESM.docx]

**Microvascular dysfunction across organs in heart failure with preserved ejection fraction: the PROSE-HFpEF case-control study**

**Supplemental Material**

Jerremy Weerts MD, PhD^1^, Blanche L.M. Schroen, PhD^1^, Arantxa Barandiarán Aizpurua MD, PhD^1^, Tos T.J.M. Berendschot, PhD^3^, Lloyd Brandts, PhD^7^, Carroll A.B. Webers, MD, PhD^3^, Sami O. Simons, MD, PhD^4^, Steven J.R. Meex, PhD^5^, Ronald Henry, MD, PhD^2^, Carla J.H. van der Kallen, PhD^2^, Hans-Peter Brunner-La Rocca, MD^1^, Christian Knackstedt MD, PhD^1^, Stephane R.B. Heymans MD, PhD^1^, Rudolf A. de Boer MD, PhD^6^, Vanessa P.M. van Empel MD, PhD^1*^, Alfons J.H.M. Houben, PhD^2*^

* Contributed equally.
1. Department of Cardiology, CARIM Cardiovascular Research Institute Maastricht, Maastricht University Medical Centre (MUMC+), Maastricht, The Netherlands

2. Department of Internal Medicine, CARIM Cardiovascular Research Institute Maastricht, Maastricht University Medical Centre (MUMC+), Maastricht, The Netherlands

3. University Eye Clinic Maastricht, Maastricht University Medical Centre (MUMC+), Maastricht, The Netherlands

4. Department of Respiratory Medicine, NUTRIM Research Institute of Nutrition and Translational Research in Metabolism, Maastricht University Medical Centre (MUMC+), Maastricht, The Netherlands

5. Department of Clinical Chemistry, CARIM School for Cardiovascular Diseases, Maastricht University Medical Centre (MUMC+), Maastricht, The Netherlands

6. Department of Cardiology, Thorax Center, Cardiovascular Institute, Erasmus MC, Rotterdam, the Netherlands
7. Department of Clinical Epidemiology and Medical Technology Assessment, Maastricht University Medical Centre (MUMC+), Maastricht, The Netherlands

Corresponding author:

Jerremy Weerts, MD, PhD

Maastricht University Medical Center+, PO Box 616, 6200 MD Maastricht, The Netherlands

Ph: +31 43 387 7097 F: +31 43 387 5104

E: jerremy.weerts@mumc.nl

Index

[Methods 4](#_Toc202545855)

[Inclusion and exclusion criteria 4](#_Toc202545856)

[Sample size calculation 5](#_Toc202545857)

[Microvascular assessments 7](#_Toc202545858)

[Protocol deviations due to the COVID-19 pandemic 9](#_Toc202545859)

[Statistical analyses 10](#_Toc202545860)

[Results 13](#_Toc202545861)

[Figure S1. Study flow diagram 13](#_Toc202545862)

[Figure S2. Moderate-to-vigorous self-reported physical activity in patients with HFpEF and 1:1 matched control individuals 14](#_Toc202545863)

[Figure S3. Data distribution of age, CRVE and UACR between groups before and after 1:1 matching 15](#_Toc202545864)

[Table S1. Linear regression models for HFpEF status of all skin flowmotion components 16](#_Toc202545865)

[Table S2. Adjusted linear regression model (model 5) of HFpEF status for each specific microvascular assessment 17](#_Toc202545866)

[Table S3. Comparisons of microvascular measurements in male and female patients with HFpEF 18](#_Toc202545867)

[Table S4. Final model with all covariates for CRVE (per µm) 19](#_Toc202545868)

[Table S5. Final model with all covariates for CRAE (per µm) 19](#_Toc202545869)

[Table S6. Final model with all covariates for retinal venular dilatation (per %) 20](#_Toc202545870)

[Table S7. Final model with all covariates for retinal arteriolar dilatation (per %) 20](#_Toc202545871)

[Table S8. Final model with all covariates for endothelial power (per log-transformed PU^2^) 21](#_Toc202545872)

[Table S9. Final model with all covariates for respiratory power (per log-transformed PU^2^) 21](#_Toc202545873)

[Table S10. Final model with all covariates for heat-induced hyperemia response (per log-transformed %) 22](#_Toc202545874)

[Table S11. Final model with all covariates for UACR (per log-transformed g/mol) 22](#_Toc202545875)

[Table S12. Linear regression of final model with HbA1c instead of diabetes mellitus status 23](#_Toc202545876)

[Table S13. Linear regression of final model with complete cases 24](#_Toc202545877)

[Table S14. Univariable adjusted logistic regression analyses for microvascular assessments on HFpEF status 25](#_Toc202545878)

[Table S15. Multivariable adjusted logistic regression analyses for microvascular assessments on HFpEF status in individuals with available NT-proBNP values 26](#_Toc202545879)

[Table S16. Clinical and microvascular characteristics in patients with HFpEF and matched controls (ratio 1:2) 27](#_Toc202545880)

[Table S17. Studies on retinal microvascular changes related to heart failure or cardiac remodelling 28](#_Toc202545881)

[References 31](#_Toc202545882)

# Methods

## Inclusion and exclusion criteria

### Patients with HFpEF

Patients with HFpEF were eligible to participate in this study based on the following inclusion criteria:

- HFpEF diagnosis based on the European Society of Cardiology (ESC) heart failure 2016 guidelines’ diagnostic criteria.^1^
- Aged 60 years or older.

Exclusion criteria for participation in patients with HFpEF:

- Inability to give informed consent.
- Contraindications for pupil dilation by ocular drips, which is needed for the primary endpoint of this study (assessed by flicker-light induced retinal vessel reactivity): a history of acute glaucoma, previous allergic reaction to ocular dilation drips, pregnancy or giving breastfeeding, current presence of intraocular oil or gas after retinal detachment.
- Contraindication for flicker-light induced retinal vessel reactivity assessment: history of photosensitive epilepsy.

### Controls

Data of controls already participating in the Maastricht Study was used. All controls signed informed consent prior to using their data for the current study.

Controls were selected based on the following inclusion criteria:

- Aged 60 years or older
- Data available of primary endpoint

Controls were excluded based on the following criteria:

- A history of HF at baseline or HF during one-year follow-up after baseline.
- Suspected severe cardiac valve disease or decreased left ventricular ejection fraction during baseline echocardiography. Or if no echocardiography was performed.
- Inclusion as HFpEF patient in the current study.

## Sample size calculation

### Choice of primary outcome microvascular assessment

Recent evidence showed that flicker-light induced retinal microvascular %-dilation reflects endothelial dysfunction and inflammation secondary to metabolic disorders,^2,3^ potentially even more so than microvascular skin tests due to impedance differences of both vascular beds.^4^ Therefore, we chose flicker-light induced retinal arteriolar microvascular %-dilation as primary endpoint.

### Expected difference

None of our microvascular assessments had been reported in HFpEF, but these tests had proven their reliability to assess microcirculation in other patient populations.^2,5^ Previous microvascular assessments in HFpEF mainly included post-occlusive reactive hyperemia assessments of a finger.^6^ Hence, we inferred the expected difference from the results of these studies. The two largest studies reported a mean difference of reactive hyperemia of -16.5% in HFpEF patients compared to controls matched at least for age, sex, and prevalence of diabetes mellitus and hypertension.^7,8^ Because retinal microvascular differences were more pronounced than skin differences in patients with diabetes mellitus versus controls (33% relative difference),^2^ we increased our expected difference of the primary outcome to 18%, corrected for the most important confounders of microvascular function.

### Sample size calculation

A ratio of HFpEF patients to controls of 1:4 was chosen to enhance statistical power for multivariable analyses,^9^ while accounting for the maximum available amount of data available from control individuals. Reference values from individuals with normal glucose metabolism^2^ were used for controls and an identical standard deviation was used for HFpEF patients. Because the Maastricht Study has been enriched by individuals with diabetes mellitus by design,^10^ diabetes mellitus is an important comorbidity in HFpEF,^11^ and control individuals were already enrolled, we aimed for doubled number of controls. This would facilitate stratified analyses by diabetes mellitus status.

| **Input Data** | | | | | |  |
| --- | --- | --- | --- | --- | --- | --- |
| Confidence Interval (2-sided) | | 95% |  | | |  |
| Power | | 80% |  | | |  |
| Ratio of sample size (Cases/Controls) | | 0.25 |  | | |  |
|  | **Controls** |  | **Cases** |  | **Difference*** |  |
| Mean | 3.4 |  |  |  | 0.612 |  |
| Standard deviation | 2.8 |  | 2.8 |  | |  |
| Variance | 7.84 |  | 7.84 |  | |  |
|  | | | | | |  |
| Sample size of Controls | | 822 | + 822 |  |  |  |
| Sample size of Cases | | 206 |  |  |  |  |
| Total sample size | | 1850 |  |  |  |  |
|  | | | | | |  |
| *Difference between the means, calculated by mean of controls divided by expected difference (3.4 * 18% = 0.612). | | | | | |  |

Results from OpenEpi, Version 3, open source calculator--SSMean

In conclusion, we aimed to include at least 1850 individuals in this study, of which 206 HFpEF patients.

## Microvascular assessments

### Participant preparation

All prospective study measurements took place during 4 hours and started in either the morning (8.00 AM) or the afternoon (1.00 PM). Study participants received study preparations by phone call and a physical letter. Preparation instructions included abstention of caffeinated drinks and smoking three hours prior study measurements, and a standardized breakfast and lunch the day of measurements. The standardized meal instructions particularly tried to prevent intake of food rich in fat, such as butter or cheese, and indicated time limits for last meal intake (6.30 AM for breakfast, 11.30 AM for lunch). To ensure optimal skin measurement results, use of skin crèmes or sunscreen before study measurements were discouraged. It was advised to take prescribed medication as taken normally.

### Measurement procedures

All measurements took place in a temperature-controlled room of 24 degrees Celsius, and care was taken to prevent that participants fell asleep during measurements.

Fifteen minutes prior retinal measurements, mydriasis was induced in both eyes by tropicamide (0.5%) and phenylephrine (2.5%) drops. Retinal measurements all took place in a light-dimmed room. Fundus photography was performed in both eyes, whereas flicker-light induced vessel analysis was performed randomly in the right or left eye. Fundus photography images were taken centered on the optic disc, centered on the macula, and the temporal field one disc diameter from the macula center. Retinal diameters were analysed with the RHINO software and calculated the central retinal arteriolar and venular equivalent based on the improved Knudtson-Hubbard formula.^12^ Retinal dilatation in response to 40 seconds of flicker light, after 50 seconds baseline assessment, was assessed with the Dynamic Vessel Analyzer hardware and software (Imedos Systems GmbH, Jena, Germany) while participants were instructed to focus on the tip of a fixed needle inside the retinal camera. Arteriolar and venular segments that were straight for approximately 1.5 millimetre in length and were located 0.5 to 2.0 disc diameters from the margin of the optic disc in the temporal section were analysed.^12,13^ Retinal dilation was calculated as mean diameter increase during 40 seconds flicker light based on two regression lines (0-10 and 10-40 seconds) divided by the mean baseline diameter of the selected vessel (arteriole or venule).

Skin flowmotion and heat-induced hyperemia response were assessed by laser Doppler flowmetry (PeriFlux 5000; Perimed AB, Järfälla, Sweden) with probes on the dorsal side of the left wrist for 25 minutes. The components of power density signals (arbitrary perfusion units, PU^2^), primarily measured from arterioles and venules, were derived from frequency domains after fast-Fourier transformation (endothelial 0.01-0.02 Hertz, neurogenic 0.02-0.06 Hertz, myogenic 0.06-0.15 Hertz, respiratory 0.15-0.40 Hertz, and cardiac 0.40-1.60 Hertz). Probes were maintained at 30 degrees Celsius. Using the same technique but different probes, a heat-induced flow response of the skin due to hyperemia assessed the percentage increase of average perfusion units during 23 minutes of 44 degrees Celsius heating compared to 2 minutes of baseline temperature.^12^

The urinary albumin-to-creatinin ratio (UACR) was assessed by albumin and creatinin levels in a urine portion.

More details of the microvascular assessment procedures and analyses can be found in a previously published methodological paper.^12^

### Covariates

The same clinical data were prospectively obtained in control individuals from The Maastricht Study through standardised questionnaires, examinations, and additional diagnostics by trained staff, as described previously.^10,14^

Physical activity was assessed using a modified Champs Activities Questionnaire for Older Adults.^10^ Total activity was calculated as the sum of the weekly duration (midpoint) of all reported activities. Moderate-to-vigorous intensity physical activity (MVPA) was calculated similarly from all activities labelled as moderate-to-vigorous, as described previously.^15^

## Protocol deviations due to the COVID-19 pandemic

The current study was in its recruitment phase (83 patients included) when the Coronavirus Disease 2019 (COVID-19) pandemic had major impact on Dutch healthcare. Several nationally enforced lockdowns and research facilities closures delayed inclusions and the pre-defined interim analysis for statistical power evaluations. The interim analysis of the primary endpoint included 103 HFpEF patients and 2140 controls (all available data of controls meeting in-& exclusion criteria). An absolute difference of -0.08 [95%CI -0.75 – 0.59] % flicker-light induced retinal dilatation was found between groups after confounder adjustment (age, sex, systolic blood pressure, body mass index, serum HbA1c and haemoglobin, and smoking status), yielding a relative adjusted-difference of 4% instead of the expected 18%. Showing significant statistical difference for these observations required very large sample sizes, as depicted below.

| **Input Data – extrapolation the observed difference** | | | | | |  |
| --- | --- | --- | --- | --- | --- | --- |
| Confidence Interval (2-sided) | | 95% |  | | |  |
| Power | | 80% |  | | |  |
| Ratio of sample size (Cases/Controls) | | 0.125 |  | | |  |
|  | **Controls** |  | **Cases** |  | **Difference** |  |
| Mean | 2.15 |  |  |  | 0.08 |  |
| Standard deviation | 3.05 |  | 3.08 |  | |  |
| Variance | 9.30 |  | 9.49 |  | |  |
|  | | | | | |  |
| Sample size of Controls | | 104,471 |  |  |  |  |
| Sample size of Cases | | 13,059 |  |  |  |  |
| Total sample size | | 117,530 |  |  |  |  |
|  | | | | | |  |

*Results from OpenEpi, Version 3, open source calculator--SSMean*

Similarly, assuming the same standard deviations as observed in the interim analyses, but with an expected difference of 18% resulted in unfeasible sample size requirements (559 patients and 4,465 control individuals). Larger sample sizes were calculated than what was anticipated, mainly due to much lower values of the control group than reported previously in younger individuals.^2^ Interpretations of the primary endpoint’s result were not deemed different with more inclusions, so the study inclusion was halted.

## Statistical analyses

### Clinical characteristics

To facilitate interpretation of sex-specific differences, microvascular assessment results were also compared between male and female patients with HFpEF using an (unadjusted) independent T-test or Mann-Whitney U test, as appropriate.

### Missing data

Multiple imputation by chained equations using fully conditional specification with predictive mean matching was performed for variables used as dependent input for the linear regression models. Imputations included 50 iterations of 20 imputations and were stratified for patients with HFpEF and control individuals. Indicating input variables included diabetes mellitus (0% missing), coronary artery disease, retinopathy, NT-proBNP, medication use, and time of microvascular assessments. Percentages of missing data were 0% (age and sex), 0.1% (BMI), 0.6% (smoking status), 0.7% (eGFR), 1.1% (HbA1c), 1.7% (Haemoglobin), 6.4% (systolic blood pressure), and 9.9% (MVPA).

### Linear regression

Linear regression instead of case-control matched analyses was pre-defined as primary analyses to retain the most statistical power. Models were constructed with continuous rather than categorical variables wherever possible to retain the most physiological information and statistical power. Continuous variables not meeting linear regression assumptions were log-transformed, are reported as such, and all met the assumptions thereafter.

### Sensitivity analyses

Sensitivity analyses were performed to evaluate the robustness of the linear regression results, including 1) linear regression *model 5* (model 4 + weekly hours of MVPA), because lower physical activity can be a cause (confounder) or a consequence (mediator) of HFpEF, which, in the case of being a mediator, might introduce bias, 2) linear regression models with serum HbA1c instead of DM status, 3) linear regression analyses using a complete case analysis, 4) linear regression analyses excluding outliers (defined as >3 absolute standard deviations), 5) logistic regression analyses using HFpEF (yes/no) as dependent variable and results of microvascular assessments as independent variable reporting Odds Ratio (OR) with 95% CI (**Supplemental Methods Logistic regression**), and 6) differences between patients with HFpEF and control individuals after case-control matching using nearest neighbour matching for age, sex, diabetes mellitus status, hypertension, and BMI with a ratio of 1:1 and 1:2. To assess the effect of HFpEF on UACR after correction for estimated renal function, *model 5b* was constructed including model 4 with the addition of estimated glomerular filtration rate (eGFR) based on the Chronic Kidney Disease Epidemiology Collaboration (CKD-EPI) equation.

### Logistic regression

Logistic regression analyses using HFpEF (yes/no) as dependent variable and results of microvascular assessments as independent variable reporting Odds Ratio (OR) with 95% CI. This included binomial logistic regression for HFpEF status with the same covariates as *model 4*. Microvascular assessments with a p-value <0.10 remained in the final multivariable model. To assess the association between microvascular markers and HFpEF after adjustment for biomarkers used in clinical care to diagnose HFpEF, NT-proBNP was added to logistic regression *model 5c* (including all clinical factors from model 4).

# Results

## Figure S1. Study flow diagram


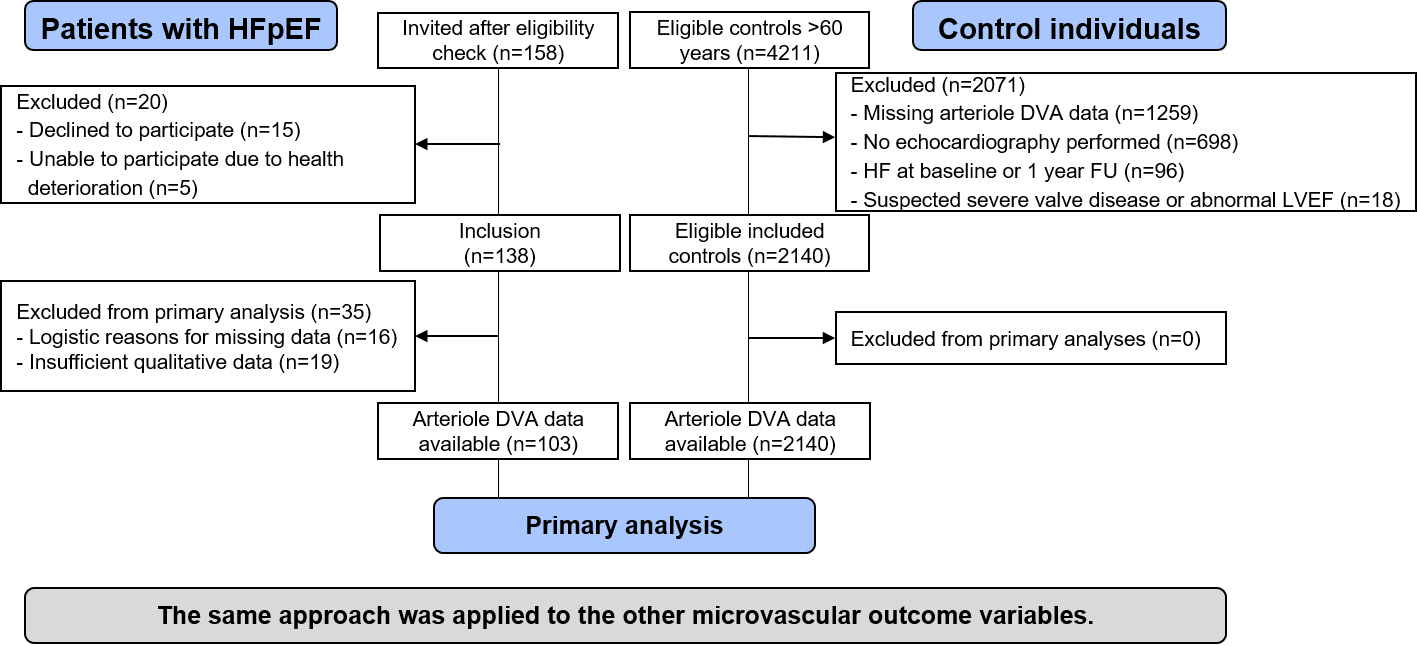


Flow diagram of subject inclusion and primary analysis of one of the microvascular assessments. DVA, retinal dynamic vessel analysis; FU, follow-up; HF, heart failure; HFpEF, heart failure with preserved ejection fraction; LVEF, left ventricular ejection fraction.

## Figure S2. Moderate-to-vigorous self-reported physical activity in patients with HFpEF and 1:1 matched control individuals


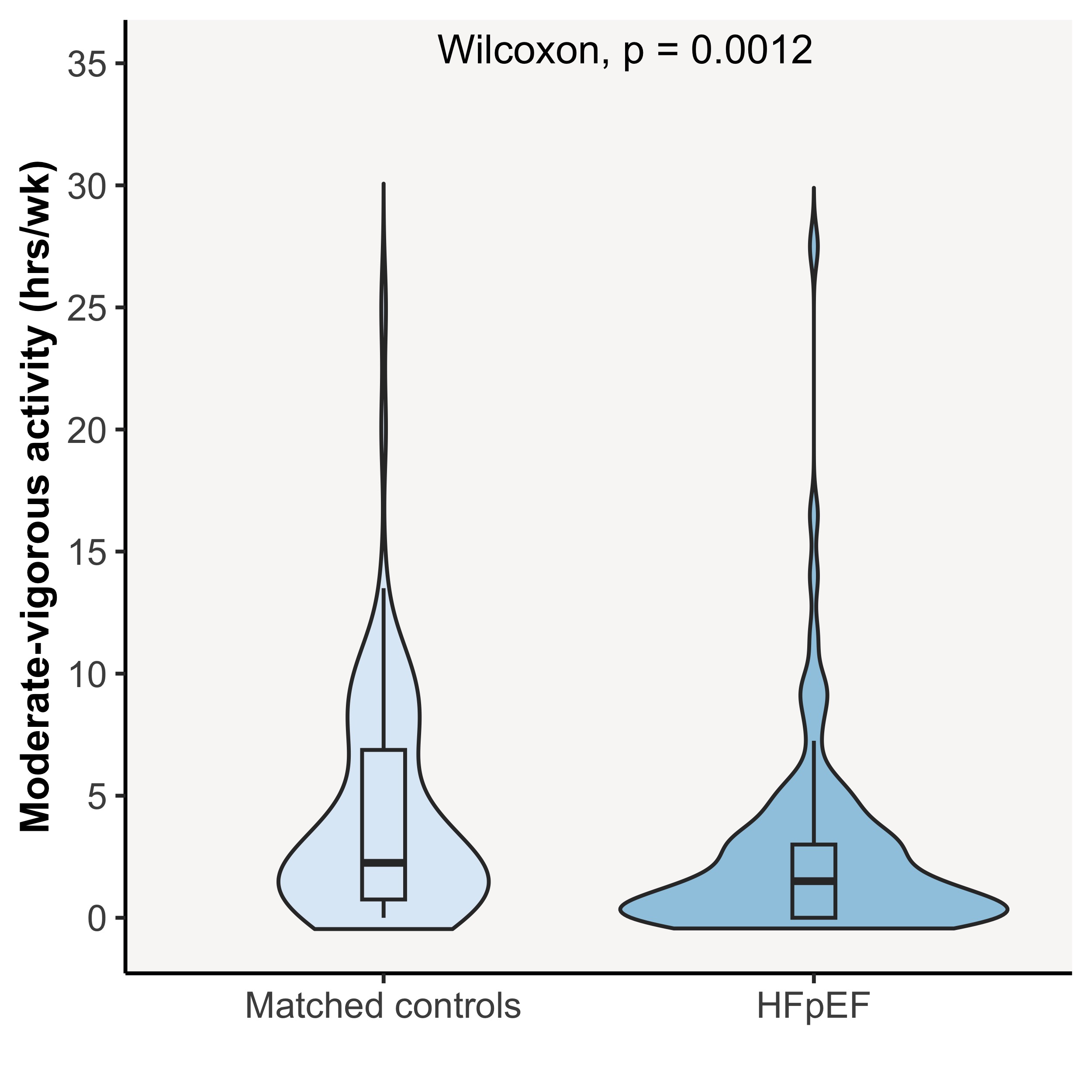


## Figure S3. Data distribution of age, CRVE and UACR between groups before and after 1:1 matching


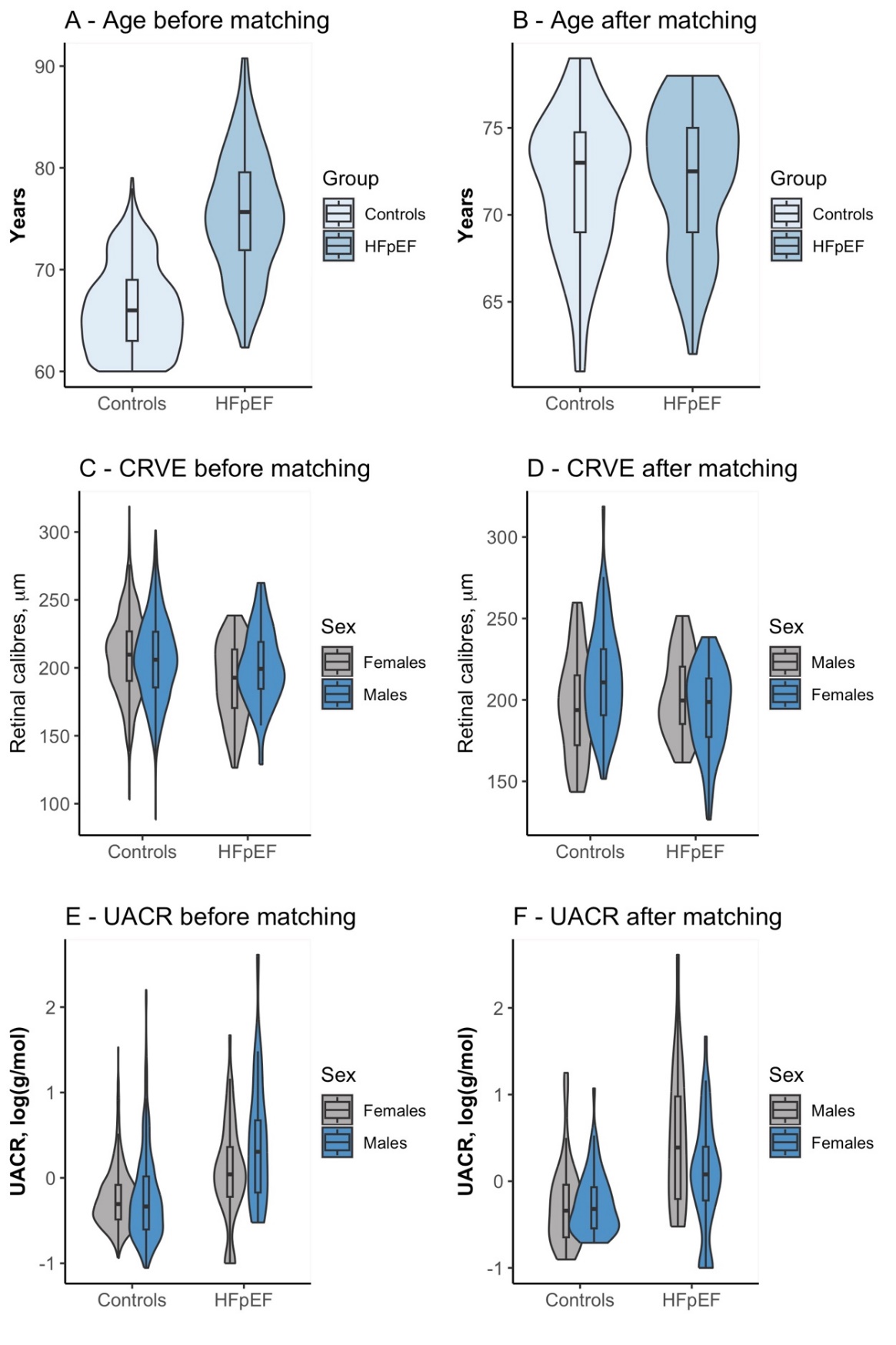


Case-control matching between patients with HFpEF and control individuals was performed using nearest neighbour matching for age, sex, diabetes mellitus status, hypertension, and BMI with a ratio of 1:1.

## Table S1. Linear regression models for HFpEF status of all skin flowmotion components

| **Variable** | **Β** | **95%CI** | **SE β** | **Standardized β*** | **p-value** |
| --- | --- | --- | --- | --- | --- |
| **Endothelial power, PU² (log-transformed)** |  |  |  |  |  |
| Model 1 | -0.13 | -0.25 - -0.01 | 0.06 | -0.044 | 0.031 |
| Model 4 (final) | -0.09 | -0.23 - 0.05 | 0.07 | -0.027 | 0.181 |
| **Myogenic power, PU² (log-transformed)** |  |  |  |  |  |
| Model 1 | 0.09 | -0.04-0.22 | 0.07 | 0.032 | 0.173 |
| Model 4 (final) | 0.06 | -0.10-0.21 | 0.08 | 0.022 | 0.721 |
| **Neurogenic power, PU² (log-transformed)** |  |  |  |  |  |
| Model 1 | -0.07 | -0.18-0.05 | 0.06 | -0.022 | 0.264 |
| Model 4 (final) | -0.06 | -0.19-0.08 | 0.07 | -0.014 | 0.428 |
| **Respiratory power, PU² (log-transformed)** |  |  |  |  |  |
| Model 1 | 0.21 | 0.08 - 0.34 | 0.07 | 0.075 | 0.001 |
| Model 4 (final) | 0.05 | -0.11 - 0.20 | 0.08 | 0.022 | 0.555 |
| **Cardiac power, PU² (log-transformed)** |  |  |  |  |  |
| Model 1 | 0.11 | -0.01-0.22 | 0.06 | 0.033 | 0.076 |
| Model 4 (final) | -0.03 | -0.17-0.10 | 0.07 | -0.014 | 0.652 |
| **Total power, PU² (log-transformed)** | | | | | |
| Model 1 | -0.07 | -0.18-0.04 | 0.06 | -0.023 | 0.231 |
| Model 4 (final) | -0.06 | -0.19-0.08 | 0.07 | -0.015 | 0.390 |
| Legend: * of original data model. Abbreviation: PU, (arbitrary) perfusion units. | | | | | |

## Table S2. Adjusted linear regression model (model 5) of HFpEF status for each specific microvascular assessment

| **Variable** | **Β** | **95%CI** | **SE β** | **Standardized β*** | **p-value** |
| --- | --- | --- | --- | --- | --- |
| CRVE, μm | -7.8 | -13.8 - -1.8 | 3.1 | -0.058 | 0.011 |
| CRAE, μm | -3.4 | -7.2 - 0.4 | 2.0 | -0.048 | 0.081 |
| Retinal venular dilatation, % | -0.21 | -0.73 - 0.32 | 0.27 | -0.023 | 0.440 |
| Retinal arteriolar dilatation, % | -0.15 | -0.83 - 0.53 | 0.35 | -0.013 | 0.661 |
| Endothelial power, PU² (log-transformed) | -0.08 | -0.22 - 0.06 | 0.07 | -0.022 | 0.260 |
| Respiratory power, PU² (log-transformed) | 0.05 | -0.10 - 0.20 | 0.08 | 0.022 | 0.490 |
| Heat-induced hyperemia response, % (log-transformed) | 0.02 | -0.05 - 0.09 | 0.03 | 0.011 | 0.569 |
| Urinary albumin-to-creatinine ratio (UACR), g/mol (log-transformed) | 0.54 | 0.43 - 0.65 | 0.06 | 0.284 | <0.001 |
| UACR model 5b^#^, g/mol (log-transformed) | 0.52 | 0.41 - 0.63 | 0.06 | 0.282 | <0.001 |
| Legend: * of original data model. Model 5 consisted of: age, sex, diabetes mellitus, systolic blood pressure, body mass index, haemoglobin, smoking status, and hours/week moderate to vigorous physical activity. ^#^ Model 5b was applied to UACR for sensitivity analyses, which consisted of the same variables as model 4, but with addition of estimated glomerular filtration (eGFR) rate according to CKD-EPI equation instead of physical activity. Abbreviations as in Table 2. | | | | | |

## Table S3. Comparisons of microvascular measurements in male and female patients with HFpEF

| **Variable** | **Male patients** | **Female patients** | **p-value** |
| --- | --- | --- | --- |
| Retinal arteriolar dilatation, % | 1.9±2.3% | 2.0±3.1% | 0.910 |
| Retinal venular dilatation, % | 3.2±2.0% | 3.7±2.3% | 0.262 |
| CRAE, μm | 129.3±18.3 | 130.7±19.2 | 0.696 |
| CRVE, μm | 200.7±27.8 | 190.8±28.0 | 0.061 |
| Endothelial power, PU² | 46,684 [17,019-121,537] | 29,214 [11,007-69,360] | 0.106 |
| Myogenic power, PU² | 6,485 [3,238-20,763] | 4,337 [1,481-16,338] | 0.107 |
| Neurogenic power, PU² | 24,116 [10,425-67,817] | 15,580 [5,035-47,480] | 0.125 |
| Respiratory power, PU² | 3,941 [1,316-13,070] | 2,189 [724-7,234] | 0.060 |
| Cardiac power, PU² | 2,002 [958-6617] | 1,657 [539-6,611] | 0.354 |
| Total power, PU² | 93,075 [42,388-225,418] | 59,649 [19,695-177,532] | 0.079 |
| Heat-induced hyperemia response, % | 1,101 [590-1,553] | 1,163 [707-1,739] | 0.459 |
| Urinary albumin-to-creatinine ratio (UACR), g/mol | 2.05 [0.63-4.98] | 1.10 [0.60-2.40] | 0.059 |
| Legend/abbreviations: CRAE, central retinal arteriolar equivalent; CRVE, central retinal venular equivalent; PU, (arbitrary) perfusion units. | | | |

## Table S4. Final model with all covariates for CRVE (per µm)

| **Variable** | **Β** | **95%CI** | **Standardized β*** | **p-value** |
| --- | --- | --- | --- | --- |
| HFpEF status | -8.07 | -14.06--2.08 | -0.055 | 0.008 |
| Age (per 1 year) | -0.73 | -1.02--0.43 | -0.111 | <0.001 |
| Female sex | 4.08 | 1.13-7.02 | 0.069 | 0.007 |
| Systolic blood pressure (per 1 mmHg) | -0.07 | -0.16-0.02 | -0.042 | 0.142 |
| Body mass index (per 1 kg/m2) | 0.62 | 0.31-0.93 | 0.081 | <0.001 |
| Diabetes mellitus | 0.24 | -2.79-3.26 | 0.016 | 0.879 |
| Haemoglobin (per 1 mmol/l) | 2.21 | 0.18-4.24 | 0.057 | 0.033 |
| Never smoked | Ref | Ref | Ref | Ref |
| Previous smoker | 3.44 | 0.69-6.20 | 0.059 | 0.014 |
| Current smoker | 8.42 | 3.82-13.02 | 0.087 | <0.001 |
| Legend: * of original data model. Abbreviations: Ref, reference. | | | | |

## Table S5. Final model with all covariates for CRAE (per µm)

| **Variable** | **Β** | **95%CI** | **Standardized β*** | **p-value** |
| --- | --- | --- | --- | --- |
| HFpEF status | -3.49 | -7.30-0.33 | -0.045 | 0.073 |
| Age (per 1 year) | -0.48 | -0.67--0.30 | -0.109 | <0.001 |
| Female sex | 4.35 | 2.47-6.22 | 0.115 | <0.001 |
| Systolic blood pressure (per 1 mmHg) | -0.16 | -0.22--0.10 | -0.132 | <0.001 |
| Body mass index (per 1 kg/m2) | 0.24 | 0.04-0.44 | 0.042 | 0.017 |
| Diabetes mellitus | 2.13 | 0.21-4.06 | 0.060 | 0.030 |
| Haemoglobin (per 1 mmol/l) | 0.04 | -1.26-1.34 | 0.003 | 0.952 |
| Never smoked | Ref | Ref | Ref | Ref |
| Previous smoker | -0.41 | -2.15-1.34 | -0.002 | 0.650 |
| Current smoker | 2.77 | -0.16-5.70 | 0.053 | 0.064 |
| Legend/abbreviations: as in Table S4. | | | | |

## Table S6. Final model with all covariates for retinal venular dilatation (per %)

| **Variable** | **Β** | **95%CI** | **Standardized β*** | **p-value** |
| --- | --- | --- | --- | --- |
| HFpEF status | -0.23 | -0.75-0.30 | -0.019 | 0.392 |
| Age (per 1 year) | -0.01 | -0.03-0.02 | -0.001 | 0.693 |
| Female sex | 0.55 | 0.31-0.79 | 0.108 | <0.001 |
| Systolic blood pressure (per 1 mmHg) | 0.003 | -0.004-0.01 | 0.013 | 0.391 |
| Body mass index (per 1 kg/m2) | -0.02 | -0.04-0.01 | -0.022 | 0.210 |
| Diabetes mellitus | -0.27 | -0.52--0.03 | -0.049 | 0.029 |
| Haemoglobin (per 1 mmol/l) | 0.10 | -0.07-0.26 | 0.025 | 0.244 |
| Never smoked | Ref | Ref | Ref | Ref |
| Previous smoker | 0.03 | -0.19-0.25 | 0.015 | 0.792 |
| Current smoker | -0.34 | -0.71-0.03 | -0.040 | 0.069 |
| Legend/abbreviations: as in Table S4. | | | | |

## Table S7. Final model with all covariates for retinal arteriolar dilatation (per %)

| **Variable** | **Β** | **95%CI** | **Standardized β*** | **p-value** |
| --- | --- | --- | --- | --- |
| HFpEF status | -0.18 | -0.86-0.49 | -0.015 | 0.593 |
| Age (per 1 year) | -0.05 | -0.08--0.02 | -0.066 | 0.001 |
| Female sex | -0.16 | -0.46-0.14 | -0.032 | 0.296 |
| Systolic blood pressure (per 1 mmHg) | 0.01 | -0.003-0.02 | 0.033 | 0.184 |
| Body mass index (per 1 kg/m2) | -0.002 | -0.03-0.03 | -0.02 | 0.885 |
| Diabetes mellitus | -0.58 | -0.88--0.27 | -0.089 | <0.001 |
| Haemoglobin (per 1 mmol/l) | 0.02 | -0.19-0.22 | 0.001 | 0.858 |
| Never smoked | Ref | Ref | Ref | Ref |
| Previous smoker | -0.08 | -0.35-0.20 | 0.001 | 0.587 |
| Current smoker | -0.58 | -1.04--0.12 | -0.052 | 0.014 |
| Legend/abbreviations: as in Table S4. | | | | |

## Table S8. Final model with all covariates for endothelial power (per log-transformed PU^2^)

| **Variable** | **Β** | **95%CI** | **Standardized β*** | **p-value** |
| --- | --- | --- | --- | --- |
| HFpEF status | -0.09 | -0.23-0.04 | -0.027 | 0.181 |
| Age (per 1 year) | 0.01 | -0.001-0.01 | 0.040 | 0.071 |
| Female sex | -0.21 | -0.28--0.14 | -0.149 | <0.001 |
| Systolic blood pressure (per 1 mmHg) | 0.000 | -0.002-0.003 | 0.017 | 0.648 |
| Body mass index (per 1 kg/m2) | -0.02 | -0.02--0.01 | -0.106 | <0.001 |
| Diabetes mellitus | 0.01 | -0.06-0.08 | 0.002 | 0.774 |
| Haemoglobin (per 1 mmol/l) | -0.01 | -0.06-0.04 | -0.011 | 0.768 |
| Never smoked | Ref | Ref | Ref | Ref |
| Previous smoker | -0.03 | -0.10-0.03 | -0.026 | 0.333 |
| Current smoker | 0.11 | 0.002-0.23 | 0.048 | 0.046 |
| Legend/abbreviations: as in Table S4. | | | | |

## Table S9. Final model with all covariates for respiratory power (per log-transformed PU^2^)

| **Variable** | **Β** | **95%CI** | **Standardized β*** | **p-value** |
| --- | --- | --- | --- | --- |
| HFpEF status | 0.05 | -0.11-0.20 | 0.022 | 0.555 |
| Age (per 1 year) | 0.03 | 0.02-0.04 | 0.178 | <0.001 |
| Female sex | -0.12 | -0.20--0.04 | -0.073 | 0.002 |
| Systolic blood pressure (per 1 mmHg) | 0.001 | -0.001-0.004 | 0.027 | 0.246 |
| Body mass index (per 1 kg/m2) | -0.02 | -0.03--0.01 | -0.112 | <0.001 |
| Diabetes mellitus | 0.02 | -0.06-0.01 | 0.005 | 0.672 |
| Haemoglobin (per 1 mmol/l) | -0.02 | -0.08-0.03 | -0.028 | 0.377 |
| Never smoked | Ref | Ref | Ref | Ref |
| Previous smoker | -0.01 | -0.08-0.07 | -0.005 | 0.835 |
| Current smoker | 0.19 | 0.07-0.32 | 0.071 | 0.002 |
| Legend/abbreviations: as in Table S4. | | | | |

## Table S10. Final model with all covariates for heat-induced hyperemia response (per log-transformed %)

| **Variable** | **Β** | **95%CI** | **Standardized β*** | **p-value** |
| --- | --- | --- | --- | --- |
| HFpEF status | 0.02 | -0.05-0.08 | 0.016 | 0.605 |
| Age (per 1 year) | -0.002 | -0.01-0.002 | -0.032 | 0.336 |
| Female sex | 0.15 | 0.11-0.18 | 0.235 | <0.001 |
| Systolic blood pressure (per 1 mmHg) | 0.000 | -0.001-0.001 | -0.018 | 0.471 |
| Body mass index (per 1 kg/m2) | -0.001 | -0.004-0.003 | -0.026 | 0.676 |
| Diabetes mellitus | -0.06 | -0.10--0.03 | -0.080 | 0.001 |
| Haemoglobin (per 1 mmol/l) | -0.004 | -0.03-0.02 | 0.000 | 0.756 |
| Never smoked | Ref | Ref | Ref | Ref |
| Previous smoker | -0.01 | -0.04-0.03 | -0.021 | 0.762 |
| Current smoker | -0.09 | -0.15--0.04 | -0.094 | 0.001 |
| Legend/abbreviations: as in Table S4. | | | | |

## Table S11. Final model with all covariates for UACR (per log-transformed g/mol)

| **Variable** | **Β** | **95%CI** | **Standardized β*** | **p-value** |
| --- | --- | --- | --- | --- |
| HFpEF status | 0.56 | 0.45-0.66 | 0.258 | <0.001 |
| Age (per 1 year) | 0.003 | -0.003-0.01 | 0.034 | 0.289 |
| Female sex | -0.07 | -0.13--0.01 | -0.079 | 0.017 |
| Systolic blood pressure (per 1 mmHg) | 0.004 | 0.002-0.01 | 0.135 | <0.001 |
| Body mass index (per 1 kg/m2) | 0.000 | -0.01-0.01 | -0.004 | 0.931 |
| Diabetes mellitus | 0.19 | 0.13-0.25 | 0.167 | <0.001 |
| Haemoglobin (per 1 mmol/l) | -0.04 | -0.08--0.001 | -0.065 | 0.043 |
| Never smoked | Ref | Ref | Ref | Ref |
| Previous smoker | 0.05 | -0.001-0.11 | 0.048 | 0.055 |
| Current smoker | 0.11 | 0.02-0.21 | 0.062 | 0.015 |
| Legend/abbreviations: as in Table S4. | | | | |

## Table S12. Linear regression of final model with HbA1c instead of diabetes mellitus status

| **Variable** | **Β** | **95%CI** | **SE β** | **Standardized β*** | **p-value** |
| --- | --- | --- | --- | --- | --- |
| **CRVE, μm** | -8.0 | -13.9 - -2.0 | 3.0 | -0.059 | 0.009 |
| *interaction between HFpEF status and sex* |  |  |  |  | *0.023* |
| stratified: males | 1.3 | -8.8 - 11.4 | 5.1 | -0.004 | 0.801 |
| stratified: females | -13.7 | -21.4 - -6.2 | 3.9 | -0.115 | <0.001 |
| *interaction between HFpEF status and HbA1c* |  |  |  |  | *0.716* |
| **CRAE, μm** | -3.8 | -7.6 - -0.3 | 1.9 | -0.045 | 0.048 |
| *interaction between HFpEF status and sex* |  |  |  |  | *0.481* |
| *interaction between HFpEF status and HbA1c* |  |  |  |  | *0.166* |
| **Retinal venular dilatation, %** | -0.2 | -0.7 - 0.3 | 0.3 | -0.012 | 0.492 |
| *interaction between HFpEF status and sex* |  |  |  |  | *0.955* |
| *interaction between HFpEF status and HbA1c* |  |  |  |  | *0.948* |
| **Retinal arteriolar dilatation, %** | -0.1 | -0.8 - 0.6 | 0.3 | -0.003 | 0.806 |
| *interaction between HFpEF status and sex* |  |  |  |  | *0.737* |
| *interaction between HFpEF status and HbA1c* |  |  |  |  | *0.692* |
| **Endothelial power, PU² (log-transformed)** | -0.1 | -0.2 - 0.04 | 0.1 | -0.030 | 0.170 |
| *interaction between HFpEF status and sex* |  |  |  |  | *0.957* |
| *interaction between HFpEF status and HbA1c* |  |  |  |  | *0.078* |
| stratified: no diabetes mellitus | -0.1 | -0.2 - 0.1 | 0.1 | -0.017 | 0.475 |
| stratified: yes diabetes mellitus | -0.4 | -0.7 - -0.1 | 0.2 | -0.111 | 0.014 |
| **Respiratory power, PU² (log-transformed)** | 0.04 | -0.1 - 0.2 | 0.1 | 0.022 | 0.560 |
| *interaction between HFpEF status and sex* |  |  |  |  | *0.232* |
| *interaction between HFpEF status and HbA1c* |  |  |  |  | *0.222* |
| **Heat-induced hyperemia response, % (log-transformed)** | 0.03 | -0.04 - 0.1 | 0.03 | 0.027 | 0.428 |
| *interaction between HFpEF status and sex* |  |  |  |  | *0.142* |
| *interaction between HFpEF status and HbA1c* |  |  |  |  | *0.643* |
| **Urinary albumin-to-creatinine ratio (UACR), g/mol (log-transformed)** | 0.5 | 0.4 - 0.6 | 0.1 | 0.253 | <0.001 |
| *interaction between HFpEF status and sex* |  |  |  |  | *0.003* |
| stratified: males | 0.7 | 0.5 - 0.9 | 0.1 | 0.53 | <0.001 |
| stratified: females | 0.5 | 0.4 - 0.6 | 0.1 | 0.306 | <0.001 |
| *interaction between HFpEF status and HbA1c* |  |  |  |  | *0.865* |
| Legend: * of original data model. Final model (4) included: HFpEF status, corrected for age, sex, HbA1c, systolic blood pressure, body mass index, haemoglobin, smoking status. Italic p-values represent p_interaction_. | | | | | |

## Table S13. Linear regression of final model with complete cases

| **Variable** | **Β** | **95%CI** | **SE β** | **Standardized β** | **p-value** |
| --- | --- | --- | --- | --- | --- |
| **CRVE, μm** | -7.14 | -13.53 - -0.75 | 3.26 | -0.055 | 0.028 |
| *interaction between HFpEF status and sex* |  |  |  |  | *0.046* |
| stratified: males | 1.55 | -9.23 – 12.34 | 5.50 | 0.009 | 0.778 |
| stratified: females | -12.41 | -20.88 - -4.60 | 4.15 | -0.119 | 0.002 |
| *interaction between HFpEF status and DM* |  |  |  |  | *0.095* |
| stratified: no DM | -9.56 | -16.84 - -2.27 | 3.72 | -0.079 | 0.010 |
| stratified: yes DM | 1.08 | -12.52 - 14.69 | 6.93 | 0.007 | 0.876 |
| **CRAE, μm** | -3.77 | -7.85 - 0.31 | 2.08 | -0.045 | 0.070 |
| *interaction between HFpEF status and sex* |  |  |  |  | *0.823* |
| *interaction between HFpEF status and DM* |  |  |  |  | *0.523* |
| **Retinal venular dilatation, %** | -0.23 | -0.79 - 0.34 | 0.29 | -0.019 | 0.434 |
| *interaction between HFpEF status and sex* |  |  |  |  | *0.793* |
| *interaction between HFpEF status and DM* |  |  |  |  | *0.357* |
| **Retinal arteriolar dilatation, %** | -0.24 | -0.97 - 0.50 | 0.37 | -0.015 | 0.528 |
| *interaction between HFpEF status and sex* |  |  |  |  | *0.834* |
| *interaction between HFpEF status and DM* |  |  |  |  | *0.541* |
| **Endothelial power, PU² (log-transformed)** | -0.07 | -0.22 - 0.07 | 0.08 | -0.027 | 0.332 |
| *interaction between HFpEF status and sex* |  |  |  |  | *0.904* |
| *interaction between HFpEF status and DM* |  |  |  |  | *0.012* |
| stratified: no DM | -0.05 | -0.22 - 0.13 | 0.09 | -0.017 | 0.611 |
| stratified: yes DM | -0.35 | -0.68 - -0.03 | 0.16 | -0.111 | 0.031 |
| **Respiratory power, PU² (log-transformed)** | 0.07 | -0.10 - 0.23 | 0.08 | 0.022 | 0.424 |
| *interaction between HFpEF status and sex* |  |  |  |  | *0.196* |
| *interaction between HFpEF status and DM* |  |  |  |  | *0.060* |
| stratified: no DM | 0.08 | -0.11 - 0.27 | 0.10 | 0.027 | 0.412 |
| stratified: yes DM | -0.19 | -0.55 - 0.17 | 0.18 | -0.053 | 0.299 |
| **Heat-induced hyperemia response, % (log-transformed)** | 0.02 | -0.05 - 0.10 | 0.04 | 0.016 | 0.558 |
| *interaction between HFpEF status and sex* |  |  |  |  | *0.063* |
| stratified: males | 0.11 | -0.02 - 0.23 | 0.06 | 0.062 | 0.091 |
| stratified: females | -0.02 | -0.11 - 0.06 | 0.05 | -0.024 | 0.535 |
| *interaction between HFpEF status and DM* |  |  |  |  | *0.559* |
| **Urinary albumin-to-creatinine ratio (UACR), g/mol (log-transformed)** | 0.53 | 0.41 - 0.64 | 0.06 | 0.285 | <0.001 |
| *interaction between HFpEF status and sex* |  |  |  |  | *0.001* |
| stratified: males | 0.67 | 0.46 - 0.88 | 0.11 | 0.253 | <0.001 |
| stratified: females | 0.40 | 0.28 - 0.52 | 0.06 | 0.306 | <0.001 |
| *interaction between HFpEF status and DM* |  |  |  |  | *0.223* |
| Legend: All results display corrections as in model 4: HFpEF status, corrected for age, sex, DM (diabetes mellitus), systolic blood pressure, body mass index, haemoglobin, smoking status. Italic p-values represent p_interaction_. | | | | | |

## Table S14. Univariable adjusted logistic regression analyses for microvascular assessments on HFpEF status

|  | **Univariable adjusted** | | | **Univariable adjusted males** | | | **Univariable adjusted females** | | |
| --- | --- | --- | --- | --- | --- | --- | --- | --- | --- |
| **Variable** | **OR** | **95%CI** | **p-value** | **OR** | **95%CI** | **p-value** | **OR** | **95%CI** | **p-value** |
| CRVE, per μm | 0.993 | 0.985 - 1.001 | 0.083 | 1.003 | 0.990 - 1.016 | 0.627 | 0.985 | 0.973 - 0.996 | 0.007 |
| CRAE, per μm | 0.989 | 0.977 - 1.002 | 0.102 | 0.994 | 0.974 - 1.015 | 0.597 | 0.986 | 0.969 - 1.003 | 0.101 |
| Retinal venular dilatation, per % | 0.945 | 0.852 - 1.050 | 0.293 | 0.967 | 0.818 - 1.143 | 0.692 | 0.929 | 0.810 - 1.065 | 0.292 |
| Retinal arteriolar dilatation, per % | 0.992 | 0.920 - 1.069 | 0.827 | 1.008 | 0.874 - 1.161 | 0.916 | 0.983 | 0.897 - 1.078 | 0.719 |
| Endothelial power, per PU² (log-transformed) | 0.737 | 0.502 - 1.083 | 0.120 | 0.662 | 0.350 - 1.252 | 0.205 | 0.735 | 0.445 - 1.215 | 0.230 |
| Respiratory power, per PU² (log-transformed) | 1.131 | 0.818 - 1.564 | 0.456 | 1.300 | 0.747 - 2.263 | 0.353 | 1.023 | 0.669 - 1.564 | 0.917 |
| Heat-induced hyperemia response, per % (log-transformed) | 1.559 | 0.681 - 3.572 | 0.293 | 1.950 | 0.503 – 7.558 | 0.334 | 1.168 | 0.390 – 3.491 | 0.782 |
| Urinary albumin-to-creatinine ratio (UACR), per g/mol (log-transformed) | 7.748 | 4.487 - 13.379 | <0.001 | 7.168 | 3.362 – 15.286 | <0.001 | 8.436 | 3.623 – 19.640 | <0.001 |
| Adjusted for age, sex, systolic blood pressure, body mass index, diabetes mellitus, Haemoglobin, and smoking status. | | | | | | | | | |

## Table S15. Multivariable adjusted logistic regression analyses for microvascular assessments on HFpEF status in individuals with available NT-proBNP values

|  | **Multivariable adjusted** | | | **Multivariable adjusted males** | | | **Multivariable adjusted females** | | |
| --- | --- | --- | --- | --- | --- | --- | --- | --- | --- |
| **Variable** | **OR** | **95%CI** | **p-value** | **OR** | **95%CI** | **p-value** | **OR** | **95%CI** | **p-value** |
| CRVE, per μm | 0.982 | 0.970-0.995 | 0.005 | 0.995 | 0.977-1.013 | 0.593 | 0.971 | 0.951-0.991 | 0.005 |
| Urinary albumin-to-creatinine ratio (UACR), per g/mol (log-transformed) | 1.982 | 1.011-3.885 | 0.046 | 2.635 | 1.122-6.190 | 0.026 | 1.287 | 0.405-4.085 | 0.669 |
| Legend: Adjusted according to model 5c for: age, sex, systolic blood pressure, body mass index, diabetes mellitus, Haemoglobin, smoking status, and NT-proBNP. | | | | | | | | | |

## Table S16. Clinical and microvascular characteristics in patients with HFpEF and matched controls (ratio 1:2)

|  |  | | | | |  |
| --- | --- | --- | --- | --- | --- | --- |
| **Variables** | **HFpEF patients (n = 102)** | **Controls (n = 204)** | | **p-value** | |  |
| **Clinical characteristics** |  |  | |  | |  |
| Age, years | 72.5 (69, 75) | 72 (69, 74) | | 0.069 | |  |
| Female sex, n (%) | 69 (68) | 139 (68) | | 1 | |  |
| Diabetes mellitus, n (%) | 26 (25) | 56 (27) | | 0.82 | |  |
| Hypertension, n (%) | 80 (78) | 157 (77) | | 0.885 | |  |
| Body mass index, kg/m2 | 28.9 (26.2, 33.7) | 29.3 (26.6, 32.8) | | 0.801 | |  |
| **Retinal microvasculature** |  |  | |  | |  |
| Retinal calibres |  |  | |  | |  |
| CRVE, µm | 197.33 ± 25.27 | 207.6 ± 31.39 | | 0.003 | |  |
| CRAE, µm | 131.61 ± 18.92 | 135.86 ± 18.94 | | 0.073 | |  |
| Flicker-light induced vasodilation | | |  | | | |
| Venular dilation, % | 3.01 (1.92, 4.28) | 3.31 (1.97, 4.73) | | 0.258 | |  |
| Arteriolar dilation, % | 1.41 (0.01, 3.56) | 1.46 (0.28, 3.74) | | 0.655 | |  |
| **Skin microvasculature** |  |  | |  | |  |
| Vasomotion |  | |  | |  | |
| Cardiac power, PU² | 1511 (728, 6076) | 1982 (645, 4927) | | 0.899 | |  |
| Endothelial power, PU² | 37558 (12080, 74805) | 35244 (13477, 101278) | | 0.511 | |  |
| Myogenic power, PU² | 5335 (2136, 16061) | 4444 (1406, 13600.25) | | 0.615 | |  |
| Neurogenic power, PU² | 16356 (8022, 37647) | 18728 (6964, 55399) | | 0.518 | |  |
| Respiratory power, PU² | 2515 (905, 6955) | 1927 (521, 6057) | | 0.237 | |  |
| Total power, PU² | 66617 (26092, 166485) | 64531 (26779, 189712) | | 0.697 | |  |
| Heat-induced hyperemia |  |  | |  | |  |
| Heat-induced hyperemia response, % | 1230 (795, 1663) | 1027 (663, 1735) | | 0.252 | |  |
| **Renal microvasculature** |  |  | |  | |  |
| Urinary albumin-to-creatinine ratio (UACR), g/mol | 1.3 (0.6, 4.05) | 0.36 (0.20, 0.60) | | < 0.001 | |  |
| Legend: Data presented as mean ± standard deviation, median (inter-quartile ranges), or count (percentage). CRAE, central retinal arteriolar equivalent; CRVE, Central retinal venular equivalent; HFpEF, heart failure with preserved ejection fraction; PU, arbitrary perfusion unit. | | | | | |  |

## Table S17. Studies on retinal microvascular changes related to heart failure or cardiac remodelling

| **Study’s first author, year** | **Outcome** | **Study population** | **Study design** | **Method (measurement)** | **Microvascular aspect assessed** | **Result (95% CI)** | **Result adjusted for confounders** | **Sex-specified results** |
| --- | --- | --- | --- | --- | --- | --- | --- | --- |
| Cheung, 2007^16^ | LV concentric remodelling (LVH) | General population, free of cardiovascular disease (MESA) (n=4,593) | Cross-sectional | Retinography (static) | Calibres | Narrowest vs widest CRAE for LVH: OR 2.06 (1.57-2.70); Widest vs narrowest CRVE for LVH: OR 1.56 (0.82–2.96) | Age, sex, race, study center, systolic blood pressure, body mass index, smoking, antihypertensive medications, diabetes presence and duration, HbA1c, lipid profile, C-reactive protein | Both sexes: narrower CRAE associated with LVH. Females: wider CRVE associated with LVH |
| Wong, 2002^17^ | Coronary artery disease (CAD) | General population, no CAD (ARIC) (n=9,648) | Prospective | Retinography (static) | Arteriolar/venular calibres ratio (AVR) | Only sex-specified outcome | Age, race, study center, 6-year mean blood pressure, diabetes, waist-hip ratio, sports index, lipid profile, smoking, alcoho consumption, antihypertensive medication use. | Lowest vs highest AVR in females for CAD: RR 2.2 (1.0-4.6). Males: 1.1 (0.7-1.8) |
| Wong, 2005^18^ | Incident HF | General population (ARIC) (n=11,612) | Prospective | Retinography (static) | Calibres | Lowest CRAE quantile for HF: RR 1.18 (0.95-1.48). Retinopathy presence for HF: 1.96 (1.51-2.54) | Age, sex,study center, educational level, CAD, 6-year mean blood pressure, antihypertensive medication use, diabetes, glucose level, LDL, smoking, body mass index | Association of retinopathy and HF was present in both sexes. |
| Chandra, 2019^19^ | Incident HF, LVH, diastolic and systolic dysfunction | General population, free of cardiovascular disease (ARIC) (n=10,629) | Prospective | Retinography (static) | Calibres | Narrower CRAE for incident HF: HR 0.92 (0.87-0.97). Wider CRVE: 1.15 (1.09-1.20). Narrower CRAE and wider CRVE were associated with larger LV dimensions and LVH. | Age, sex, race | Female sex was decreasing in prevalence with CRAE narrowing and CRVE widening (quartiles). CRVE was stronger associated with LVH in females, and with lower systolic LV function in males. |
| Gillum, 1991^20^ | CAD | General population (n=7,710) | Cross-sectional | Fundoscopy | Increased light reflex, narrow arterioles, tortuous arterioles, and arteriovenous compression (nicking) in one or both fundi | Only sex-specified outcome | Hypertesion, lipid profile | Retinal abnormalities for CAD in females (35-54yrs): RR 6.4 (uncorrected), (55-79yrs) RR 2.4 (1.3-4.2). In males (35-54yrs): 3.1 (1.5-6.5), (55-79yrs) 1.3 (0.8-2.0) |
| Tromp, 2019^21^ | Microvascular disease | Symptomatic HF with diabetes mellitus, in Asian country (n=2,800) | Cross-sectional + outcomes | Self-reported history and medical record history | Retinopathy, neuropathy, nephropathy | Microvascular complications for HFpEF: OR 1.70 (1.15-2.50). Microvascular complications were independently associated with adverse outcomes in HFrEF and HFpEF | Age, sex, ethnicity, CAD< atrial fibrillation, stroke, peripheral artery disease, hypertension, NYHA class, duration of HF, HF medications, serum creatinine, hemoglobin, and diabetes mellitus presence, medication and duration. | Not reported |
| Nägele, 2018^22^ | Retinal microvascular dysfunction | 1) HF (n=74), 2) patients with cardiovascular risk factors without cardiovascular disease (CVRF) (n=74), 3) healthy non-smoking individuals (HC) (n=74) | Cross-sectional | Retinography (static and response to flicker-light) | Calibres, responsive dilatation | CRAE and CRVE not different between groups. Arteriolar dilatation decreased in HF. Venular and arteriolar dilatation was similar between HFpEF and HFrEF | None. Subanalyses showed correlations in HF patients with echocardiographic and blood markers mainly with retinal venular dilation. | Not reported |
| Ikram, 2005^23^ | Hypertension | General population without hypertension (n=1,900) | Prospective | Retinography (static) | Calibres | CRVE but not CRAE was narrowed in patients with prehypertesion vs. controls. Narrower CRAE and CRVE for indicent HT: OR 1.38 (1.23-1.55) and 1.17 (1.04-1.32) | Age, sex, follow-up time, body mass index, smoking, diabetes mellitus, total and HDL cholesterol, c-reactive protein, intima-media thickness | Not reported |

Legend: Most studies with heart failure patients did not include patients with HFpEF specifically, or did not report results for patients with HFpEF separately.^18,19,22^ One study included patients with HFpEF based on LVEF ≥50% and one HF decompensation episode in last 6 months (n=561).^21^

# References

1. Ponikowski P, Voors AA, Anker SD, Bueno H, Cleland JG, Coats AJ, Falk V, Gonzalez-Juanatey JR, Harjola VP, Jankowska EA, Jessup M, Linde C, Nihoyannopoulos P, Parissis JT, Pieske B, Riley JP, Rosano GM, Ruilope LM, Ruschitzka F, Rutten FH, van der Meer P, Authors/Task Force M, Document R. 2016 ESC Guidelines for the diagnosis and treatment of acute and chronic heart failure: The Task Force for the diagnosis and treatment of acute and chronic heart failure of the European Society of Cardiology (ESC). Developed with the special contribution of the Heart Failure Association (HFA) of the ESC. Eur J Heart Fail 2016;**18**(8):891-975.

2. Sorensen BM, Houben AJ, Berendschot TT, Schouten JS, Kroon AA, van der Kallen CJ, Henry RM, Koster A, Sep SJ, Dagnelie PC, Schaper NC, Schram MT, Stehouwer CD. Prediabetes and Type 2 Diabetes Are Associated With Generalized Microvascular Dysfunction: The Maastricht Study. Circulation 2016;**134**(18):1339-1352.

3. Mimoun L, Massin P, Steg G. Retinal microvascularisation abnormalities and cardiovascular risk. Arch Cardiovasc Dis 2009;**102**(5):449-56.

4. Martens RJH, Houben A, Kooman JP, Berendschot T, Dagnelie PC, van der Kallen CJH, Kroon AA, Leunissen KML, van der Sande FM, Schaper NC, Schouten J, Schram MT, Sep SJS, Sorensen BM, Henry RMA, Stehouwer CDA. Microvascular endothelial dysfunction is associated with albuminuria: the Maastricht Study. Journal of hypertension 2018;**36**(5):1178-1187.

5. Houben A, Martens RJH, Stehouwer CDA. Assessing Microvascular Function in Humans from a Chronic Disease Perspective. Journal of the American Society of Nephrology : JASN 2017;**28**(12):3461-3472.

6. Weerts J, Mourmans SGJ, Barandiaran Aizpurua A, Schroen BLM, Knackstedt C, Eringa E, Houben A, van Empel VPM. The Role of Systemic Microvascular Dysfunction in Heart Failure with Preserved Ejection Fraction. Biomolecules 2022;**12**(2).

7. Akiyama E, Sugiyama S, Matsuzawa Y, Konishi M, Suzuki H, Nozaki T, Ohba K, Matsubara J, Maeda H, Horibata Y, Sakamoto K, Sugamura K, Yamamuro M, Sumida H, Kaikita K, Iwashita S, Matsui K, Kimura K, Umemura S, Ogawa H. Incremental prognostic significance of peripheral endothelial dysfunction in patients with heart failure with normal left ventricular ejection fraction. J Am Coll Cardiol 2012;**60**(18):1778-86.

8. Yamamoto E, Hirata Y, Tokitsu T, Kusaka H, Sakamoto K, Yamamuro M, Kaikita K, Watanabe H, Hokimoto S, Sugiyama S, Maruyama T, Ogawa H. The pivotal role of eNOS uncoupling in vascular endothelial dysfunction in patients with heart failure with preserved ejection fraction. Int J Cardiol 2015;**190**:335-7.

9. Hennessy S, Bilker WB, Berlin JA, Strom BL. Factors influencing the optimal control-to-case ratio in matched case-control studies. Am J Epidemiol 1999;**149**(2):195-7.

10. Schram MT, Sep SJS, van der Kallen CJ, Dagnelie PC, Koster A, Schaper N, Henry RMA, Stehouwer CDA. The Maastricht Study: an extensive phenotyping study on determinants of type 2 diabetes, its complications and its comorbidities. European Journal of Epidemiology 2014;**29**(6):439-451.

11. Paulus WJ, Tschope C. A novel paradigm for heart failure with preserved ejection fraction: comorbidities drive myocardial dysfunction and remodeling through coronary microvascular endothelial inflammation. J Am Coll Cardiol 2013;**62**(4):263-71.

12. Li W, Schram MT, Sorensen BM, van Agtmaal MJM, Berendschot T, Webers CAB, Jansen JFA, Backes WH, Gronenschild E, Schalkwijk CG, Stehouwer CDA, Houben A. Microvascular Phenotyping in the Maastricht Study: Design and Main Findings, 2010-2018. Am J Epidemiol 2020;**189**(9):873-884.

13. Sorensen BM, Houben A, Berendschot T, Schouten J, Kroon AA, van der Kallen CJH, Henry RMA, Koster A, Reesink KD, Dagnelie PC, Schaper NC, Schalkwijk CG, Schram MT, Stehouwer CDA. Hyperglycemia Is the Main Mediator of Prediabetes- and Type 2 Diabetes-Associated Impairment of Microvascular Function: The Maastricht Study. Diabetes Care 2017;**40**(8):e103-e105.

14. Sorensen BM, van der Heide FCT, Houben A, Koster A, T TJMB, J SAGS, Kroon AA, van der Kallen CJH, Henry RMA, van Dongen M, S JPME, H HCMS, van der Berg JD, Schaper NC, Schram MT, Stehouwer CDA. Higher levels of daily physical activity are associated with better skin microvascular function in type 2 diabetes-The Maastricht Study. Microcirculation 2020;**27**(4):e12611.

15. Hekler EB, Buman MP, Haskell WL, Conway TL, Cain KL, Sallis JF, Saelens BE, Frank LD, Kerr J, King AC. Reliability and validity of CHAMPS self-reported sedentary-to-vigorous intensity physical activity in older adults. J Phys Act Health 2012;**9**(2):225-36.

16. Cheung N, Bluemke DA, Klein R, Sharrett AR, Islam FM, Cotch MF, Klein BE, Criqui MH, Wong TY. Retinal arteriolar narrowing and left ventricular remodeling: the multi-ethnic study of atherosclerosis. J Am Coll Cardiol 2007;**50**(1):48-55.

17. Wong TY, Klein R, Sharrett AR, Duncan BB, Couper DJ, Tielsch JM, Klein BEK, Hubbard LD. Retinal Arteriolar Narrowing and Risk of Coronary Heart Disease in Men and WomenThe Atherosclerosis Risk in Communities Study. JAMA 2002;**287**(9):1153-1159.

18. Wong TY, Rosamond W, Chang PP, Couper DJ, Sharrett AR, Hubbard LD, Folsom AR, Klein R. Retinopathy and risk of congestive heart failure. JAMA 2005;**293**(1):63-9.

19. Chandra A, Seidelmann SB, Claggett BL, Klein BE, Klein R, Shah AM, Solomon SD. The association of retinal vessel calibres with heart failure and long-term alterations in cardiac structure and function: the Atherosclerosis Risk in Communities (ARIC) Study. Eur J Heart Fail 2019;**21**(10):1207-1215.

20. Gillum RF. Retinal arteriolar findings and coronary heart disease. Am Heart J 1991;**122**(1, Part 1):262-263.

21. Tromp J, Lim SL, Tay WT, Teng TK, Chandramouli C, Ouwerkerk W, Wander GS, Sawhney JPS, Yap J, MacDonald MR, Ling LH, Sattar N, McMurray JJV, Richards AM, Anand I, Lam CSP, Investigators A-H. Microvascular Disease in Patients With Diabetes With Heart Failure and Reduced Ejection Versus Preserved Ejection Fraction. Diabetes Care 2019;**42**(9):1792-1799.

22. Nagele MP, Barthelmes J, Ludovici V, Cantatore S, von Eckardstein A, Enseleit F, Luscher TF, Ruschitzka F, Sudano I, Flammer AJ. Retinal microvascular dysfunction in heart failure. Eur Heart J 2018;**39**(1):47-56.

23. Ikram MK, Witteman JCM, Vingerling JR, Breteler MMB, Hofman A, de Jong PTVM. Retinal Vessel Diameters and Risk of Hypertension. Hypertension 2006;**47**(2):189-194.
